# Global, regional, and national burden of chronic kidney disease, 1990–2017: a systematic analysis for the Global Burden of Disease Study 2017

**DOI:** 10.1016/S0140-6736(20)30045-3

**Published:** 2020-02-29

**Authors:** GBD Chronic Kidney Disease Collaboration, Boris Bikbov, Caroline A Purcell, Andrew S Levey, Mari Smith, Amir Abdoli, Molla Abebe, Oladimeji M Adebayo, Mohsen Afarideh, Sanjay Kumar Agarwal, Marcela Agudelo-Botero, Elham Ahmadian, Ziyad Al-Aly, Vahid Alipour, Amir Almasi-Hashiani, Rajaa M Al-Raddadi, Nelson Alvis-Guzman, Saeed Amini, Tudorel Andrei, Catalina Liliana Andrei, Zewudu Andualem, Mina Anjomshoa, Jalal Arabloo, Alebachew Fasil Ashagre, Daniel Asmelash, Zerihun Ataro, Maha Moh'd Wahbi Atout, Martin Amogre Ayanore, Alaa Badawi, Ahad Bakhtiari, Shoshana H Ballew, Abbas Balouchi, Maciej Banach, Simon Barquera, Sanjay Basu, Mulat Tirfie Bayih, Neeraj Bedi, Aminu K Bello, Isabela M Bensenor, Ali Bijani, Archith Boloor, Antonio M Borzì, Luis Alberto Cámera, Juan J Carrero, Félix Carvalho, Franz Castro, Ferrán Catalá-López, Alex R Chang, Ken Lee Chin, Sheng-Chia Chung, Massimo Cirillo, Ewerton Cousin, Lalit Dandona, Rakhi Dandona, Ahmad Daryani, Rajat Das Gupta, Feleke Mekonnen Demeke, Gebre Teklemariam Demoz, Desilu Mahari Desta, Huyen Phuc Do, Bruce B Duncan, Aziz Eftekhari, Alireza Esteghamati, Syeda Sadia Fatima, João C Fernandes, Eduarda Fernandes, Florian Fischer, Marisa Freitas, Mohamed M Gad, Gebreamlak Gebremedhn Gebremeskel, Begashaw Melaku Gebresillassie, Birhanu Geta, Mansour Ghafourifard, Alireza Ghajar, Nermin Ghith, Paramjit Singh Gill, Ibrahim Abdelmageed Ginawi, Rajeev Gupta, Nima Hafezi-Nejad, Arvin Haj-Mirzaian, Arya Haj-Mirzaian, Ninuk Hariyani, Mehedi Hasan, Milad Hasankhani, Amir Hasanzadeh, Hamid Yimam Hassen, Simon I Hay, Behnam Heidari, Claudiu Herteliu, Chi Linh Hoang, Mostafa Hosseini, Mihaela Hostiuc, Seyed Sina Naghibi Irvani, Sheikh Mohammed Shariful Islam, Nader Jafari Balalami, Spencer L James, Simerjot K Jassal, Vivekanand Jha, Jost B Jonas, Farahnaz Joukar, Jacek Jerzy Jozwiak, Ali Kabir, Amaha Kahsay, Amir Kasaeian, Tesfaye Dessale Kassa, Hagazi Gebremedhin Kassaye, Yousef Saleh Khader, Rovshan Khalilov, Ejaz Ahmad Khan, Mohammad Saud Khan, Young-Ho Khang, Adnan Kisa, Csaba P Kovesdy, Barthelemy Kuate Defo, G Anil Kumar, Anders O Larsson, Lee-Ling Lim, Alan D Lopez, Paulo A Lotufo, Azeem Majeed, Reza Malekzadeh, Winfried März, Anthony Masaka, Hailemariam Abiy Alemu Meheretu, Tomasz Miazgowski, Andreea Mirica, Erkin M Mirrakhimov, Prasanna Mithra, Babak Moazen, Dara K Mohammad, Reza Mohammadpourhodki, Shafiu Mohammed, Ali H Mokdad, Linda Morales, Ilais Moreno Velasquez, Seyyed Meysam Mousavi, Satinath Mukhopadhyay, Jean B Nachega, Girish N Nadkarni, Jobert Richie Nansseu, Gopalakrishnan Natarajan, Javad Nazari, Bruce Neal, Ruxandra Irina Negoi, Cuong Tat Nguyen, Rajan Nikbakhsh, Jean Jacques Noubiap, Christoph Nowak, Andrew T Olagunju, Alberto Ortiz, Mayowa Ojo Owolabi, Raffaele Palladino, Mona Pathak, Hossein Poustchi, Swayam Prakash, Narayan Prasad, Alireza Rafiei, Sree Bhushan Raju, Kiana Ramezanzadeh, Salman Rawaf, David Laith Rawaf, Lal Rawal, Robert C Reiner, Aziz Rezapour, Daniel Cury Ribeiro, Leonardo Roever, Dietrich Rothenbacher, Godfrey M Rwegerera, Seyedmohammad Saadatagah, Saeed Safari, Berhe Weldearegawi Sahle, Hosni Salem, Juan Sanabria, Itamar S Santos, Arash Sarveazad, Monika Sawhney, Elke Schaeffner, Maria Inês Schmidt, Aletta Elisabeth Schutte, Sadaf G Sepanlou, Masood Ali Shaikh, Zeinab Sharafi, Mehdi Sharif, Amrollah Sharifi, Diego Augusto Santos Silva, Jasvinder A Singh, Narinder Pal Singh, Malede Mequanent M Sisay, Amin Soheili, Ipsita Sutradhar, Berhane Fseha Teklehaimanot, Berhe etsay Tesfay, Getnet Fetene Teshome, Jarnail Singh Thakur, Marcello Tonelli, Khanh Bao Tran, Bach Xuan Tran, Candide Tran Ngoc, Irfan Ullah, Pascual R Valdez, Santosh Varughese, Theo Vos, Linh Gia Vu, Yasir Waheed, Andrea Werdecker, Haileab Fekadu Wolde, Adam Belay Wondmieneh, Sarah Wulf Hanson, Tomohide Yamada, Yigizie Yeshaw, Naohiro Yonemoto, Hasan Yusefzadeh, Zoubida Zaidi, Leila Zaki, Sojib Bin Zaman, Nelson Zamora, Afshin Zarghi, Kaleab Alemayehu Zewdie, Johan Ärnlöv, Josef Coresh, Norberto Perico, Giuseppe Remuzzi, Chris J L Murray, Theo Vos

## Abstract

**Background:**

Health system planning requires careful assessment of chronic kidney disease (CKD) epidemiology, but data for morbidity and mortality of this disease are scarce or non-existent in many countries. We estimated the global, regional, and national burden of CKD, as well as the burden of cardiovascular disease and gout attributable to impaired kidney function, for the Global Burden of Diseases, Injuries, and Risk Factors Study 2017. We use the term CKD to refer to the morbidity and mortality that can be directly attributed to all stages of CKD, and we use the term impaired kidney function to refer to the additional risk of CKD from cardiovascular disease and gout.

**Methods:**

The main data sources we used were published literature, vital registration systems, end-stage kidney disease registries, and household surveys. Estimates of CKD burden were produced using a Cause of Death Ensemble model and a Bayesian meta-regression analytical tool, and included incidence, prevalence, years lived with disability, mortality, years of life lost, and disability-adjusted life-years (DALYs). A comparative risk assessment approach was used to estimate the proportion of cardiovascular diseases and gout burden attributable to impaired kidney function.

**Findings:**

Globally, in 2017, 1·2 million (95% uncertainty interval [UI] 1·2 to 1·3) people died from CKD. The global all-age mortality rate from CKD increased 41·5% (95% UI 35·2 to 46·5) between 1990 and 2017, although there was no significant change in the age-standardised mortality rate (2·8%, −1·5 to 6·3). In 2017, 697·5 million (95% UI 649·2 to 752·0) cases of all-stage CKD were recorded, for a global prevalence of 9·1% (8·5 to 9·8). The global all-age prevalence of CKD increased 29·3% (95% UI 26·4 to 32·6) since 1990, whereas the age-standardised prevalence remained stable (1·2%, −1·1 to 3·5). CKD resulted in 35·8 million (95% UI 33·7 to 38·0) DALYs in 2017, with diabetic nephropathy accounting for almost a third of DALYs. Most of the burden of CKD was concentrated in the three lowest quintiles of Socio-demographic Index (SDI). In several regions, particularly Oceania, sub-Saharan Africa, and Latin America, the burden of CKD was much higher than expected for the level of development, whereas the disease burden in western, eastern, and central sub-Saharan Africa, east Asia, south Asia, central and eastern Europe, Australasia, and western Europe was lower than expected. 1·4 million (95% UI 1·2 to 1·6) cardiovascular disease-related deaths and 25·3 million (22·2 to 28·9) cardiovascular disease DALYs were attributable to impaired kidney function.

**Interpretation:**

Kidney disease has a major effect on global health, both as a direct cause of global morbidity and mortality and as an important risk factor for cardiovascular disease. CKD is largely preventable and treatable and deserves greater attention in global health policy decision making, particularly in locations with low and middle SDI.

**Funding:**

Bill & Melinda Gates Foundation.

## Introduction

Chronic kidney disease (CKD) is an important contributor to morbidity and mortality from non-communicable diseases, and this disease should be actively addressed to meet the UN's Sustainable Development Goal target to reduce premature mortality from non-communicable diseases by a third by 2030. Treatment costs for CKD rose after the 1960s, with availability of renal replacement techniques making possible the long-term application of life-saving but costly treatment for patients with end-stage kidney disease (ESKD).^1^ The number of people receiving renal replacement therapy exceeds 2·5 million and is projected to double to 5·4 million by 2030;^2^ however, in many countries, there is a shortage of renal replacement services, and an estimated 2·3–7·1 million adults have died prematurely from lack of access to this treatment.^2^ The effect of CKD also expands well beyond the provision of renal replacement services. Large-scale, nationally representative screening programmes undertaken in the 2000s in Australia,^3^ Norway,^4^ and the USA^5^ showed that more than 10% of the adult population have markers for kidney disease. Different research groups have reviewed the prevalence of and mortality from CKD in Africa,^6^ Asia,^7^, ^8^, ^9^, ^10^ Australia,^3^ Europe,^11^ Latin America,^12^ North America,^13^ and several geographically dispersed developing countries,^14^ and confirmed the high burden of this disease. The primary cause of CKD varies by setting, with hypertension and diabetes being the most common causes,^15^ whereas factors such as HIV^16^ and exposure to toxins or heavy metals^17^ have an additional role in developing countries. In some areas of the world with especially high burdens of CKD, the cause remains unknown.^18^

**Research in context**

**Evidence before this study**

The burden of chronic kidney disease (CKD) is studied predominantly in high-income countries, mainly in terms of prevalence, quality of life, mortality, and kidney and cardiovascular complications. Even where results of large-scale national CKD screening programmes are available, many data sources report CKD estimates only for selected populations (limited by age group, geography, occupation, etc), and for many countries there are no data for CKD epidemiology. The Global Burden of Diseases, Injuries, and Risk Factors Study (GBD) is a major effort to collect and incorporate into one system all available data for 354 diseases and 84 risk factors from the published literature, registries, vital registration systems, verbal autopsies, hospital records data, etc. GBD applies comprehensive statistical modelling to produce comparable estimates of the burden at the global, regional, and national levels.

**Added value of this study**

We compiled data for CKD epidemiology by doing several systematic literature reviews in PubMed and Embase, using search queries combining “chronic kidney disease” and “prevalence”, and we retrieved publications from 1980 onwards. Data related to dialysis and kidney transplantation were extracted from annual renal replacement therapy registry reports up to 2017 and including the most recent year of available data. The results are a variety of estimates of morbidity and mortality for CKD as a direct cause of burden, and impaired kidney function as a risk factor for cardiovascular disease and gout. The statistical modelling approach enabled us to produce estimates of kidney disease burden even for countries without primary data. We showed a substantial burden of both CKD and impaired kidney function at the global level. Prevalence of CKD, in particular, is high (9·1% of the global population). Overall, CKD and its effect on cardiovascular disease resulted in 2·6 million (95% uncertainty interval 2·4 to 2·8) deaths and 35·8 million (33·7 to 38·0) disability-adjusted life-years (DALYs), with most DALYs attributable to CKD occurring in middle and low-middle Socio-demographic Index (SDI) quintiles. Importantly, CKD has continued to rise in rank among leading causes of death over the 27-year period studied, because of ageing and an increasing burden of risk factors for CKD (including diabetes and hypertension) that, together, contributed to more than half the deaths from CKD in 2017.

**Implications of all the available evidence**

The results presented here can help stakeholders form a comprehensive action plan to prevent and treat kidney disease. These actions should include increasing the awareness of CKD among the general public and health-care authorities as well as educational programmes for health-care personnel, appropriate treatment of risk factors, management of earlier stages of CKD, and development of facilities for treating patients with end-stage kidney disease. This work could be especially relevant for countries in low and middle SDI quintiles, where there is a large gap between CKD burden and provision of adequate health care.

CKD has also been recognised as a risk factor for cardiovascular disease independent of other conventional risk factors for cardiovascular disease.^19^ CKD is associated with an increased risk for cardiovascular disease mortality and is a risk multiplier in patients with hypertension and diabetes.^15^, ^20^, ^21^ Importantly, early detection and treatment of diabetes, hypertension, and CKD is possible using readily available, often inexpensive, treatments. These interventions can improve renal and cardiovascular outcomes and slow or prevent progression to ESKD.^22^, ^23^, ^24^ Despite the availability of such interventions, the burden of CKD and its related risk factors remains understudied in many areas of the world. Even in countries with available data, disease awareness is low among both the general public and health-care authorities.^25^ As a result, many countries have underdeveloped nephrology workforces or mainly focus on the provision of treatment for ESKD but not for early stages of CKD.

The Global Burden of Disease, Injuries, and Risk Factors Study (GBD), with its broad collection of data sources and statistical modelling approaches, can deliver the most comprehensive estimates of CKD burden to date.^26^, ^27^, ^28^, ^29^ We aimed to summarise GBD 2017 findings on CKD epidemiology in 195 countries, expressed in terms of incidence, prevalence, years lived with disability (YLDs), mortality, years of life lost (YLLs), and disability-adjusted life-years (DALYs). GBD also quantifies the burden of health loss due to cardiovascular disease and gout that can be attributed to kidney dysfunction. In this Article, we use the term CKD to refer to elevated urinary albumin:creatinine ratio (ACR), decreased estimated glomerular filtration rate (eGFR), dialysis, or kidney transplantation as direct causes of morbidity and mortality, whereas we use the term impaired kidney function to refer to elevated ACR or decreased eGFR without dialysis or kidney transplantation as risk factors for cardiovascular disease and gout in addition to the direct estimates of mortality and morbidity from CKD. Most published work is based on one timepoint to define elevated ACR or decreased eGFR, excluding the ability to examine the full Kidney Disease: Improving Global Outcomes (KDIGO) definition of chronicity, which includes persistence for at least 3 months. We estimated the burden from CKD and the additional burden from comorbid cardiovascular disease and gout that can be attributed to impaired kidney function. We followed the Guidelines for Accurate and Transparent Health Estimates Reporting (GATHER; appendix pp 32–33).^30^

## Methods

In GBD, all appropriate data and statistical methods are used to borrow strength from predictive covariates and geographical proximity to countries with data, so estimates can be obtained for countries and years with few or no primary data sources. Data collected based on different case-definitions or study methods are adjusted to the level they would have been at if data were obtained using a reference method or case-definition set, for each disease or risk factor. Similarly, cause-of-death data from vital registrations or verbal autopsy studies are adjusted by reassigning deaths coded to less informative International Classification of Diseases and Injuries (ICD) codes. To account for uncertainty in primary data sources, data manipulations, measurement error, and the choice of model, all entities quantified in GBD are estimated 1000 times over in the ensemble and meta-regression models, to produce final estimates with 95% uncertainty intervals (UIs), which comprise the 2·5th and 97·5th percentiles of the 1000 draws.

### Mortality

General methods for processing, standardisation, and modelling of cause-of-death data in GBD 2017 are described in the appendix (pp 2–5), and are published elsewhere.^27^ Briefly, we modelled mortality assigned to diagnostic (ICD) codes for CKD using vital registration, verbal autopsy, and surveillance system data for 1980–2017. Cause-of-death data were mapped to GBD categories based on ICD-9 and ICD-10 codes (appendix p 12). Aggregated data were age–sex split, and intermediate or poorly defined ICD codes were redistributed to a more appropriate GBD cause using regression or proportional redistribution methods.^27^ Most codes redistributed to CKD belonged to hypertension, unspecified anaemia, and heart failure.

We used the GBD Cause of Death Ensemble model (CODEm) process to produce estimates of mortality due to CKD. CODEm fits mixed-effects and spatiotemporal Gaussian process regression models using predictive covariates to estimate either the fraction of total deaths due to CKD (cause fraction) or mortality rates by location, year, age, and sex from 1980 to 2017. The final model is a weighted average of submodels, with weights assigned based on out-of-sample predictive validity. Cause fractions from CODEm models were multiplied by GBD all-cause mortality estimates to approximate the number of deaths and mortality rate due to CKD. We scaled CODEm results for all causes to be consistent with all-cause mortality estimates by country, year, age, and sex.

### Non-fatal estimation

We defined prevalent CKD as an abnormality of kidney function, indicated by low eGFR based on serum creatinine measurement, elevated ACR, or both. We modelled six categories of prevalent CKD, defined by level of eGFR and ACR or renal replacement therapy. The categories defined in GBD are CKD stages 1–2, stage 3, stage 4, and stage 5, ESKD on maintenance dialysis, and kidney transplantation. The GBD definition of CKD differs from that presented in the KDIGO 2012 Clinical Practice Guidelines,^22^ because the GBD definition uses only one measurement of eGFR and ACR and, thus, does not formally fit the KDIGO duration requirement of abnormalities for more than 3 months. The GBD definition also does not take into account markers of kidney damage other than ACR, since these are often not reported in epidemiological studies used to estimate disease occurrence. The criteria used to define CKD categories in GBD, and how these entities correspond with KDIGO guidelines, are presented in table 1.

**Table 1:** CKD staging definitions in the GBD study and correspondence with KDIGO categories

	**Definition**	**KDIGO category**
CKD stages 1 and 2	eGFR ≥60 mL/min per 1·73 m^2^ and ACR ≥30 mg/g (not including kidney transplant recipients)	G1–G2, A2–A3 (not including kidney transplant recipients)
CKD stage 3	eGFR 30–59 mL/min per 1·73 m^2^ (not including kidney transplant recipients)	G3a–G3b, A1–A3 (not including kidney transplant recipients)
CKD stage 4	eGFR 15–29 mL/min per 1·73 m^2^ (not including kidney transplant recipients)	G4, A1–A3 (not including kidney transplant recipients)
CKD stage 5	eGFR <15 mL/min per 1·73 m^2^ (not including kidney transplant recipients or patients treated by dialysis)	G5, A1–A3 (not including kidney transplant recipients or patients treated by dialysis)
ESKD	Kidney transplant recipients and patients treated by dialysis	Not applicable (classified according to G category, with use of a D or T modifier)

CKD=chronic kidney disease. GBD=Global Burden of Diseases, Injuries, and Risk Factors Study. KDIGO=Kidney Disease: Improving Global Outcomes. eGFR=estimated glomerular filtration rate. ACR=albumin:creatinine ratio. ESKD=end-stage kidney disease. G=glomerular filtration rate. A=albuminuria. D=dialysis. T=transplant.

Building on past data collection efforts for GBD,^31^ we updated the systematic review of the literature for cross-sectional or cohort studies reporting population-representative prevalence of each stage of CKD based on serum creatinine measurements or eGFR and ACR values. New data for dialysis and transplantation were largely obtained from national registries for ESKD. We included 216 unique data sources for the prevalence of CKD stages 1–2, stage 3, stage 4, and stage 5 and 193 sources of data for incidence and prevalence of renal replacement therapy. A full list of sources used in the CKD non-fatal estimation process is available through the Global Health Data Exchange.

We modelled each stage of CKD separately in DisMod-MR 2.1, which is a Bayesian mixed-effects meta-regression tool developed specifically for GBD by the Institute for Health Metrics and Evaluation (Seattle, WA, USA). DisMod uses input data for several epidemiological parameters, including prevalence, incidence, remission rate, and excess mortality rate, to make consistent estimates of each parameter. We also ran a DisMod-MR 2.1 model for the prevalence of CKD stages 3–5 combined and scaled estimates for the prevalence of CKD stage 3, stage 4, and stage 5 to sum to the prevalence of stages 3–5 combined for each country–year–age–sex group.

The Chronic Kidney Disease Epidemiology Collaboration (CKD-EPI) equation was designated as the reference equation for estimating GFR in adults aged 18 years and older.^32^ We adjusted data reporting eGFR with the Modification of Diet in Renal Disease (MDRD) formula by a fixed ratio to the level of data reporting prevalence with the CKD-EPI equation.^33^ The CKD stages 1–2 model included fixed-effects to adjust input data using alternate ACR thresholds (17 mg/g, 20 mg/g, or 25 mg/g) to define elevated urinary ACR. Studies using dipstick tests to assess proteinuria were not used.

CKD is one of the causes of anaemia estimated in GBD. Anaemia prevalence was approximated for all causes combined and then attributed to underlying causes. Methods related to the cause-specific attribution of anaemia prevalence have been described elsewhere.^26^, ^34^

### Estimates of CKD by cause

We estimated the burden of CKD for each of five causes: type 1 diabetes, type 2 diabetes, glomerulonephritis, hypertension, and a residual category of other and unspecified causes. For CKD stages 1–2, stage 3, stage 4, and stage 5, we estimated stage-specific distributions of causes. We used available laboratory and ICD-coded diagnosis data from Geisinger Health System,^35^ a health maintenance organisation in central and northern Pennsylvania, to identify patients with CKD stages 1–2, stage 3, stage 4, or stage 5 not on renal replacement therapy. For every individual with CKD, we used ICD codes for primary renal diseases to map individuals to GBD cause groupings (appendix p 12). Individuals with CKD but no ICD code for a primary renal disease were classified as having CKD of uncertain cause. We ran a multinomial logistic regression including sex and a non-linear term for age to predict the probability of each cause by age and sex for each stage of CKD.

We used data from ESKD registries to model the proportion of ESKD and CKD mortality attributable to each cause. Registry data were extracted to represent the proportion of cases of ESKD attributable to each modelled cause of CKD, excluding cases with unknown or missing cause. We ran a DisMod-MR 2.1 model for each cause. Results from the five cause proportion models were then scaled to sum to 1 for each country–year–age–sex and applied to CKD cause-of-death data and ESKD prevalence and incidence estimates.

### DALY estimation

We multiplied estimates of deaths due to CKD by estimates of standard life expectancy by age, to produce estimates of YLLs for CKD.^27^ Non-fatal CKD estimates were separated into 15 unique sequelae dependent on CKD stage and anaemia severity (appendix pp 6–13). We calculated YLDs for each CKD sequela, multiplying the prevalence of each sequela by its corresponding disability weight.^26^ We summed YLDs and YLLs for each CKD cause to estimate DALYs. We quantified uncertainty around all estimates by taking 1000 draws from the distributions of sampling error, residual error, and corrections for measurement error. Inherent to the CODEm methods is the additional uncertainty from model choice. Final 95% UIs were calculated.

### Socio-demographic Index

Socio-demographic Index (SDI) is a summary measure of development, created as a composite of a country's total fertility rate for women younger than 25 years, educational attainment, and lag-distributed income per capita. Methods of SDI development and computation are detailed elsewhere.^36^ The expected relation between SDI and DALY rates was determined by fitting a Gaussian process regression on estimates for all locations from 1980 to 2017.

### Risk estimation

We estimated the burden of CKD stages 1–2, stage 3, stage 4, and stage 5 not including renal replacement therapy, termed impaired kidney function (table 1), as a risk factor for ischaemic heart disease, stroke, peripheral vascular disease, and gout. For the estimation of risk factors in GBD we use a counterfactual approach rather than the categorical assignment to causes. One cannot categorically assign a case or death from ischaemic heart disease to impaired kidney function while there is evidence for an elevated risk of ischaemic heart disease in the presence of impaired kidney function. In the counterfactual approach we answer the question, what would the number of cases or deaths of ischaemic heart disease have been if no one in the population had impaired kidney function? The difference between the counterfactual and observed levels of ischaemic heart disease is what is being attributed to the risk factor. To capture the full range of health loss resulting from decreased eGFR and elevated ACR, we also attributed 100% of CKD prevalence to impaired kidney function, such that all CKD burden was also considered as burden attributable to impaired kidney function exposure. We set the theoretical minimum risk exposure level (TMREL) at an ACR of 30 mg/g or lower and eGFR of 60 mL/min per 1·73 m^2^ or higher, given that this population is at the lowest risk for cardiovascular disease events.^19^, ^21^, ^37^, ^38^, ^39^, ^40^, ^41^, ^42^, ^43^, ^44^, ^45^

Relative risk values for cardiovascular disease outcomes were calculated by the Chronic Kidney Disease Prognosis Consortium, a research consortium composed of investigators representing several cohorts, including population-level cohorts with prospective data.^21^, ^46^, ^47^, ^48^, ^49^, ^50^, ^51^, ^52^, ^53^, ^54^, ^55^ Relative risk was first calculated within each cohort, then a pooled analysis of cohort-level relative risks was done using a random-effects meta-analysis. Gout relative risk was calculated by a random-effects meta-analysis of four studies,^56^, ^57^, ^58^, ^59^ considering only CKD stages 3–5 indicating increased risk of gout.

Exposure, relative risk, and TMREL were used to calculate the fraction of fatal and non-fatal burden attributable to exposure to impaired kidney function (appendix pp 14–15).

### Role of the funding source

The funder had no role in study design, data collection, data analysis, data interpretation, or writing of the report. All authors had full access to all data in the study and had final responsibility for the decision to submit for publication.

## Results

### Global prevalence

Globally in 2017, there were 697·5 million (95% UI 649·2 to 752·1) cases of CKD (table 2). Almost a third of patients with CKD lived in two countries, China (132·3 million [95% UI 121·8 to 143·7] cases) and India (115·1 million [106·8 to 124·1] cases). Bangladesh, Brazil, Indonesia, Japan, Mexico, Nigeria, Pakistan, Russia, the USA, and Vietnam had more than 10 million cases of CKD each. 79 of 195 countries included in GBD had more than 1 million prevalent cases of CKD in 2017.

**Table 2:** Prevalence and deaths for chronic kidney disease in 2017, and percentage change of age-standardised rates by location, 1990–2017

	**Prevalence (95% UI)**			**Deaths (95% UI)**		
	Count, 2017	Age-standardised rate per 100 000, 2017	Percentage change in age-standardised rates between 1990 and 2017	Count, 2017	Age-standardised rate per 100 000, 2017	Percentage change in age-standardised rates between 1990 and 2017
**Global**	**697 509 472 (649 209 403 to 752 050 655)**	**8724 (8124 to 9403)**	**1·2% (−1·1 to 3·5)**	**1 230 168 (1 195 114 to 1 258 829)**	**15·9 (15·5 to 16·3)**	**2·8% (−1·5 to 6·3)**
Low SDI	77 774 182 (72 385 752 to 84 063 152)	9216 (8586 to 9953)	3·5% (0·5 to 7·1)	129 592 (122 760 to 148 511)	19·6 (18·5 to 22·5)	−18·7% (−24·3 to −11·5)
Low-middle SDI	130 543 398 (121 443 210 to 140 950 835)	9699 (9026 to 10 459)	4·8% (2·2 to 7·6)	264 227 (248 849 to 277 138)	23·6 (22·2 to 24·7)	−4·7% (−11·4 to 1·9)
Middle SDI	200 995 222 (186 451 768 to 216 402 786)	8921 (8299 to 9622)	2·9% (0·7 to 4·9)	415 257 (387 925 to 426 580)	20·3 (19·0 to 20·8)	6·6% (−0·3 to 10·7)
High-middle SDI	158 186 168 (147 025 105 to 170 499 238)	9093 (8464 to 9784)	−4·6% (−6·7 to −2·4)	173 695 (168 779 to 178 805)	10·4 (10·1 to 10·7)	−7·9% (−12·9 to −4·4)
High SDI	127 352 466 (118 614 944 to 137 500 134)	6804 (6340 to 7331)	−4·4% (−7·3 to −1·8)	243 425 (238 219 to 249 279)	9·5 (9·3 to 9·7)	4·1% (2·0 to 6·4)
**East Asia**	**139 556 765 (128 479 977 to 151 557 920)**	**7201 (6677 to 7766)**	**−5·9% (−7·9 to −4·1)**	**189 323 (174 072 to 197 062)**	**10·2 (9·4 to 10·6)**	**−17·9% (−28·7 to −12·6)**
China	132 324 202 (121 756 611 to 143 737 211)	7180 (6658 to 7747)	−6·1% (−8·1 to −4·3)	175 891 (160 601 to 183 366)	10·0 (9·2 to 10·4)	−19·0% (−30·2 to −13·6)
North Korea	2 233 310 (2 069 070 to 2 414 053)	7300 (6796 to 7885)	2·5% (−0·2 to 5·4)	3639 (3088 to 4240)	12·3 (10·4 to 14·3)	10·3% (−9·6 to 36·8)
Taiwan (province of China)	2 751 072 (2 552 629 to 2 961 715)	8145 (7557 to 8770)	−4·9% (−8·0 to −1·7)	6743 (6319 to 7164)	17·4 (16·3 to 18·5)	−15·5% (−21·9 to −8·8)
**Southeast Asia**	**69 598 036 (64 285 483 to 75 118 675)**	**10 802 (10 029 to 11 635)**	**6·0% (3·8 to 8·3)**	**134 459 (127 712 to 142 283)**	**24·5 (23·3 to 25·8)**	**−4·3% (−9·8 to 2·1)**
Cambodia	1 262 506 (1 165 067 to 1 366 871)	9580 (8872 to 10 366)	1·7% (−2·0 to 5·7)	1838 (1621 to 2097)	17·1 (15·3 to 19·5)	−33·3% (−43·4 to −21·5)
Indonesia	27 232 922 (25 084 990 to 29 398 099)	11 164 (10 372 to 12 008)	7·6% (5·5 to 10·0)	35 446 (33 322 to 38 551)	17·3 (16·2 to 19·3)	−16·5% (−23·8 to −8·9)
Laos	573 411 (529 796 to 620 592)	10 781 (10 001 to 11 628)	1·9% (−1·3 to 5·2)	1046 (881 to 1211)	24·8 (21·3 to 28·6)	−38·8% (−49·9 to −26·0)
Malaysia	3 187 367 (2 920 099 to 3 476 760)	11 079 (10 178 to 12 020)	9·7% (5·9 to 13·8)	4731 (4285 to 5249)	21·1 (19·1 to 23·2)	−3·6% (−15·0 to 13·5)
Maldives	41 258 (37 840 to 44 813)	10 021 (9311 to 10 796)	−8·0% (−10·8 to −5·2)	68 (62 to 75)	25·5 (23·1 to 28·1)	−54·8% (−61·2 to −47·8)
Mauritius	218 092 (201 865 to 235 917)	13 768 (12 789 to 14 903)	19·1% (14·1 to 24·1)	1070 (978 to 1158)	67·0 (61·4 to 72·3)	54·4% (39·1 to 68·8)
Myanmar	5 258 275 (4 859 659 to 5 677 295)	10 658 (9909 to 11 506)	2·6% (−0·5 to 5·4)	12 026 (10 523 to 13 747)	28·4 (25·0 to 32·5)	−31·9% (−44·8 to −16·5)
Philippines	9 317 802 (8 615 652 to 10 028 481)	11 049 (10 263 to 11 879)	13·2% (10·2 to 16·1)	34 051 (30 042 to 38 555)	50·3 (44·5 to 56·4)	108·9% (83·0 to 137·8)
Sri Lanka	2 745 171 (2 543 226 to 2 967 595)	11 428 (10 612 to 12 348)	15·9% (12·3 to 19·4)	4512 (3727 to 5288)	19·9 (16·7 to 23·1)	−17·0% (−30·9 to −1·9)
Seychelles	12 354 (11 436 to 13 289)	11 094 (10 330 to 11 897)	3·5% (0·8 to 6·4)	41 (38 to 45)	41·4 (38·0 to 44·7)	24·4% (9·1 to 38·6)
Thailand	9 560 638 (8 760 453 to 10 414 024)	10 292 (9461 to 11 174)	−3·9% (−7·2 to −0·3)	21 922 (19 297 to 24 420)	23·3 (20·5 to 25·9)	−12·6% (−23·6 to −0·7)
Timor-Leste	93 556 (86 875 to 101 038)	10 362 (9623 to 11 208)	3·6% (0·4 to 7·1)	147 (121 to 175)	19·4 (16·3 to 22·8)	−26·1% (−39·9 to −9·8)
Vietnam	10 003 107 (9 262 628 to 10 787 589)	10 107 (9407 to 10 887)	5·4% (1·9 to 9·1)	17 384 (15 393 to 19 609)	20·6 (18·2 to 23·0)	−20·2% (−32·6 to −5·0)
**Oceania**	**1 097 010** (**1 007 760 to 1 187 872)**	**12 329 (11 465 to 13 266)**	**9·0% (6·5 to 11·4)**	**2900 (2500 to 3318)**	**45·2 (40·0 to 50·0)**	**22·3% (2·9 to 39·3)**
American Samoa	6328 (5837 to 6843)	13 217 (12 268 to 14 234)	11·1% (8·0 to 14·4)	24 (21 to 26)	64·6 (57·2 to 71·3)	58·5% (27·2 to 83·5)
Federated States of Micronesia	10 839 (10 011 to 11 776)	12 990 (12 071 to 14 015)	13·5% (10·4 to 16·7)	39 (31 to 46)	62·6 (51·3 to 72·9)	42·6% (12·9 to 74·3)
Fiji	119 629 (110 015 to 129 696)	14 399 (13 355 to 15 577)	8·8% (6·0 to 12·0)	278 (243 to 316)	44·7 (39·2 to 50·3)	32·2% (4·9 to 60·0)
Guam	21 526 (19 937 to 23 259)	12 252 (11 374 to 13 220)	14·3% (11·2 to 17·4)	60 (53 to 66)	35·5 (31·2 to 38·9)	82·0% (45·3 to 108·7)
Kiribati	11 496 (10 579 to 12 475)	13 285 (12 293 to 14 311)	9·0% (6·0 to 12·1)	28 (22 to 32)	42·5 (34·5 to 48·9)	29·1% (−0·8 to 53·1)
Marshall Islands	5295 (4861 to 5739)	12 311 (11 398 to 13 279)	8·5% (5·6 to 11·2)	20 (16 to 23)	65·4 (54·8 to 75·6)	25·6% (5·6 to 46·3)
Northern Mariana Islands	6379 (5849 to 6965)	12 283 (11 407 to 13 247)	9·9% (7·2 to 12·8)	18 (16 to 20)	43·3 (38·6 to 48·3)	22·2% (2·0 to 45·8)
Papua New Guinea	745 077 (682 404 to 809 149)	11 936 (11 088 to 12 856)	10·0% (6·8 to 13·0)	1964 (1605 to 2356)	42·5 (35·7 to 49·6)	18·3% (−3·2 to 41·0)
Samoa	19 031 (17 609 to 20 486)	12 455 (11 562 to 13 418)	10·5% (7·8 to 13·2)	56 (48 to 65)	46·6 (39·5 to 53·2)	47·3% (20·0 to 78·4)
Solomon Islands	53 882 (49 662 to 58 673)	12 402 (11 496 to 13 391)	10·9% (8·0 to 13·5)	149 (129 to 173)	43·0 (38·0 to 48·8)	6·9% (−14·9 to 32·9)
Tonga	11 679 (10 801 to 12 621)	13 491 (12 508 to 14 570)	10·0% (7·1 to 13·0)	38 (31 to 45)	50·9 (41·8 to 59·6)	38·7% (11·8 to 63·6)
Vanuatu	25 397 (23 508 to 27 562)	12 378 (11 488 to 13 336)	12·6% (10·0 to 15·6)	67 (51 to 85)	43·5 (34·2 to 53·6)	37·4% (5·9 to 76·5)
**Central Asia**	**8 648 124** (**8 016 509 to 9 331 529)**	**10 604 (9867 to 11 401)**	**7·5% (5·5 to 9·4)**	**9506 (9049 to 10 015)**	**13·1 (12·5 to 13·8)**	**60·9% (53·3 to 68·9)**
Armenia	434 331 (403 778 to 468 881)	11 302 (10 521 to 12 145)	10·7% (6·3 to 15·9)	430 (407 to 452)	10·6 (10·1 to 11·1)	67·8% (56·2 to 80·7)
Azerbaijan	1 101 700 (1 015 266 to 1 187 536)	10 937 (10 133 to 11 809)	8·9% (6·0 to 12·0)	1169 (1024 to 1298)	14·2 (12·2 to 15·7)	83·4% (50·9 to 108·2)
Georgia	559 200 (519 403 to 604 881)	10 810 (10 028 to 11 660)	12·1% (7·6 to 16·8)	785 (732 to 834)	13·6 (12·7 to 14·4)	128·5% (111·7 to 147·0)
Kazakhstan	1 774 266 (1 642 487 to 1 917 077)	10 093 (9350 to 10 878)	1·2% (−1·3 to 3·7)	1485 (1394 to 1585)	9·3 (8·7 to 9·9)	35·2% (26·8 to 44·3)
Kyrgyzstan	482 340 (447 058 to 520 716)	9482 (8822 to 10 237)	−0·5% (−3·9 to 2·9)	451 (422 to 483)	9·1 (8·6 to 9·8)	15·4% (6·6 to 24·6)
Mongolia	269 339 (248 417 to 290 867)	10 050 (9331 to 10 833)	−7·1% (−10·5 to −3·5)	328 (294 to 391)	15·3 (13·8 to 17·3)	−62·1% (−67·1 to −49·0)
Tajikistan	656 378 (609 283 to 709 617)	10 093 (9403 to 10 903)	1·8% (−1·7 to 5·7)	641 (569 to 701)	11·2 (9·8 to 12·3)	17·2% (2·3 to 29·6)
Turkmenistan	473 830 (438 811 to 510 957)	10 949 (10 200 to 11 786)	8·8% (5·7 to 11·7)	632 (588 to 681)	16·0 (14·9 to 17·2)	76·1% (62·2 to 91·5)
Uzbekistan	2 896 741 (2 684 400 to 3 127 941)	10 993 (10 244 to 11 818)	14·2% (10·7 to 17·5)	3584 (3184 to 4020)	15·9 (14·3 to 17·7)	104·5% (81·3 to 128·4)
**Central Europe**	**13 951 402** (**12 930 450 to 15 136 020)**	**7659 (7115 to 8282)**	**−2·7% (−6·2 to 1·4)**	**16 284 (15 806 to 16 706)**	**7·5 (7·3 to 7·7)**	**−21·2% (−23·6 to −18·9)**
Albania	272 017 (251 454 to 295 588)	7259 (6756 to 7864)	−1·1% (−5·2 to 3·1)	363 (302 to 447)	9·2 (7·7 to 11·2)	−17·2% (−30·2 to −1·4)
Bosnia and Herzegovina	427 229 (394 850 to 464 061)	8273 (7655 to 8943)	5·9% (1·3 to 10·4)	604 (550 to 662)	10·5 (9·6 to 11·5)	−0·4% (−10·1 to 9·9)
Bulgaria	981 339 (909 610 to 1 061 687)	8000 (7420 to 8630)	2·6% (−0·7 to 5·9)	1447 (1346 to 1557)	10·1 (9·4 to 10·8)	45·7% (35·3 to 57·2)
Croatia	562 778 (520 865 to 610 153)	7779 (7206 to 8390)	1·2% (−3·1 to 6·2)	829 (776 to 888)	8·8 (8·2 to 9·4)	35·3% (25·6 to 46·0)
Czech Republic	1 378 623 (1 278 611 to 1 497 741)	7998 (7442 to 8628)	−2·5% (−7·0 to 2·8)	1257 (1173 to 1348)	5·9 (5·5 to 6·4)	−28·4% (−33·6 to −22·4)
Hungary	1 323 316 (1 226 092 to 1 433 403)	8204 (7596 to 8881)	1·0% (−2·4 to 4·5)	1553 (1457 to 1646)	7·6 (7·2 to 8·1)	34·4% (26·2 to 44·1)
Montenegro	71 678 (66 286 to 77 704)	8118 (7528 to 8773)	−1·5% (−5·0 to 2·4)	127 (113 to 141)	13·1 (11·7 to 14·5)	−1·2% (−12·3 to 11·0)
North Macedonia	253 249 (235 058 to 274 801)	8308 (7720 to 8982)	2·4% (−1·7 to 6·9)	309 (280 to 341)	9·4 (8·5 to 10·3)	−11·3% (−20·5 to −0·8)
Poland	4 335 349 (3 981 687 to 4 770 568)	7271 (6702 to 7943)	−6·0% (−12·9 to 0·9)	3442 (3238 to 3669)	4·8 (4·5 to 5·1)	−50·1% (−53·5 to −46·2)
Romania	2 313 736 (2 124 485 to 2 516 663)	7292 (6716 to 7930)	−4·9% (−7·9 to −1·9)	3043 (2852 to 3223)	8·0 (7·6 to 8·5)	−34·1% (−38·5 to −29·7)
Serbia	1 142 513 (1 063 208 to 1 237 929)	8421 (7846 to 9069)	−0·5% (−4·1 to 3·3)	2386 (1982 to 2607)	14·8 (12·4 to 16·1)	18·8% (7·3 to 32·0)
Slovakia	623 048 (576 652 to 676 236)	7736 (7188 to 8341)	−3·1% (−7·2 to 1·3)	713 (653 to 775)	8·1 (7·4 to 8·8)	−34·9% (−41·5 to −27·6)
Slovenia	266 527 (247 205 to 289 578)	7581 (7056 to 8179)	−1·1% (−5·7 to 3·2)	213 (195 to 232)	4·4 (4·0 to 4·8)	−30·2% (−36·7 to −23·1)
**Eastern Europe**	**38 150 170** (**35 346 449 to 41 409 966)**	**12 408 (11 509 to 13 389)**	**3·0% (0·1 to 6·5)**	**15 734 (15 390 to 16 112)**	**4·8 (4·7 to 4·9)**	**−11·2% (−13·3 to −9·0)**
Belarus	1 558 671 (1 440 559 to 1 695 898)	11 089 (10 287 to 12 028)	−1·6% (−6·6 to 4·7)	485 (451 to 525)	3·1 (2·9 to 3·3)	−32·7% (−37·4 to −27·1)
Estonia	258 859 (240 037 to 279 019)	12 058 (11 180 to 13 022)	3·6% (0·7 to 6·5)	268 (237 to 303)	8·9 (7·9 to 10·1)	156·9% (124·7 to 194·1)
Latvia	386 621 (358 442 to 419 031)	11 899 (11 041 to 12 884)	5·3% (0·8 to 9·6)	259 (232 to 291)	6·2 (5·5 to 6·9)	41·5% (25·5 to 59·6)
Lithuania	533 970 (494 198 to 578 929)	11 328 (10 507 to 12 282)	1·1% (−2·9 to 5·7)	253 (235 to 270)	4·3 (4·0 to 4·6)	−6·1% (−13·5 to 1·8)
Moldova	587 124 (542 018 to 635 358)	11 355 (10 494 to 12 260)	1·2% (−2·1 to 5·4)	207 (196 to 219)	3·8 (3·6 to 4·0)	−3·7% (−9·7 to 3·2)
Russia	26 981 655 (24 997 909 to 29 311 266)	12 832 (11 918 to 13 878)	4·5% (1·4 to 8·1)	11 361 (11 135 to 11 621)	5·1 (5·0 to 5·2)	−14·7% (−16·8 to −12·7)
Ukraine	7 843 270 (7 253 666 to 8 469 940)	11 571 (10 707 to 12 495)	−1·6% (−4·8 to 2·1)	2900 (2750 to 3066)	4·1 (3·8 to 4·3)	−4·3% (−9·6 to 1·9)
**High-income Asia-Pacific**	**27 550 513** (**25 730 339 to 29 608 610)**	**8098 (7569 to 8699)**	**−7·5% (−10·3 to −5·2)**	**42 468 (40 550 to 45 192)**	**7·6 (7·3 to 8·1)**	**−41·1% (−43·5 to −37·6)**
Brunei	40 907 (37 231 to 44 776)	10 323 (9524 to 11 169)	−8·7% (−11·5 to −5·7)	70 (64 to 77)	26·0 (23·7 to 28·6)	−13·2% (−27·1 to 0·2)
Japan	21 411 356 (19 946 798 to 23 210 020)	8404 (7838 to 9068)	−5·9% (−8·6 to −3·3)	35 709 (33 921 to 38 263)	7·5 (7·1 to 8·0)	−40·3% (−43·0 to −36·3)
South Korea	5 451 810 (5 023 911 to 5 877 265)	7103 (6560 to 7659)	−9·2% (−14·5 to −5·6)	6095 (5630 to 6562)	7·7 (7·1 to 8·2)	−40·6% (−45·8 to −35·1)
Singapore	646 440 (598 329 to 700 408)	9333 (8659 to 10 090)	−8·8% (−10·9 to −6·6)	594 (555 to 639)	9·0 (8·4 to 9·7)	−36·7% (−41·5 to −31·4)
**Australasia**	**2 919 853** (**2 708 028 to 3 164 634)**	**6964 (6471 to 7518)**	**−0·7% (−4·5 to 3·3)**	**5228 (4833 to 5656)**	**9·2 (8·5 to 10·0)**	**8·8% (−0·7 to 18·8)**
Australia	2 471 845 (2 295 264 to 2 676 433)	6982 (6497 to 7530)	−0·9% (−4·7 to 3·3)	4455 (4068 to 4886)	9·2 (8·3 to 10·1)	6·1% (−4·8 to 17·6)
New Zealand	448 008 (415 420 to 484 747)	6859 (6375 to 7436)	0·2% (−4·0 to 4·9)	773 (732 to 819)	9·4 (8·9 to 10·0)	21·6% (13·5 to 29·8)
**Western Europe**	**41 976 625** (**38 902 049 to 45 587 058)**	**5446 (5069 to 5894)**	**−5·0% (−7·4 to −2·6)**	**90 450 (87 105 to 93 977)**	**7·8 (7·5 to 8·1)**	**−0·7% (−4·4 to 3·6)**
Andorra	6496 (6040 to 7053)	5243 (4883 to 5674)	0·8% (−3·7 to 5·5)	6 (5 to 7)	3·6 (3·1 to 4·2)	−17·9% (−30·8 to −3·0)
Austria	857 140 (791 470 to 932 601)	5557 (5173 to 6011)	5·3% (1·7 to 9·0)	2754 (2583 to 2939)	12·4 (11·6 to 13·2)	128·0% (111·7 to 145·4)
Belgium	1 101 945 (1 017 463 to 1 201 817)	5642 (5238 to 6088)	−1·4% (−4·9 to 1·9)	2097 (1942 to 2265)	7·2 (6·7 to 7·7)	−29·4% (−34·9 to −23·4)
Cyprus	107 744 (100 226 to 116 301)	6108 (5693 to 6585)	−6·4% (−9·6 to −3·0)	256 (190 to 288)	13·2 (9·9 to 14·9)	−23·5% (−34·1 to −12·6)
Denmark	552 944 (509 440 to 602 169)	5816 (5400 to 6285)	4·9% (1·8 to 8·1)	968 (903 to 1039)	7·7 (7·2 to 8·3)	117·1% (101·1 to 135·2)
Finland	563 542 (521 219 to 612 719)	5761 (5354 to 6220)	−4·5% (−8·6 to −0·2)	569 (528 to 611)	4·0 (3·7 to 4·3)	18·5% (8·9 to 28·5)
France	6 077 964 (5 589 293 to 6 632 818)	5242 (4858 to 5697)	−1·7% (−6·0 to 2·5)	9279 (8650 to 10 025)	4·9 (4·6 to 5·3)	−25·8% (−31·8 to −18·7)
Germany	9 046 875 (8 323 728 to 9 881 743)	5687 (5256 to 6173)	−4·7% (−7·7 to −1·4)	26 754 (24 215 to 29 510)	11·3 (10·2 to 12·4)	45·0% (30·3 to 61·3)
Greece	1 097 180 (1 007 649 to 1 203 008)	5342 (4962 to 5806)	−5·3% (−9·1 to −1·1)	3582 (3333 to 3859)	12·0 (11·2 to 12·9)	−30·0% (−35·3 to −24·1)
Iceland	25 097 (23 178 to 27 266)	5235 (4848 to 5703)	0·4% (−4·8 to 6·0)	27 (25 to 29)	4·3 (4·0 to 4·6)	−1·2% (−10·0 to 8·5)
Ireland	394 543 (365 205 to 426 816)	5985 (5552 to 6485)	−4·0% (−8·4 to 0·7)	579 (533 to 629)	7·5 (6·9 to 8·2)	−24·8% (−31·8 to −18·1)
Israel	654 367 (606 819 to 708 940)	6246 (5810 to 6757)	0·6% (−2·1 to 3·3)	2242 (2088 to 2407)	17·7 (16·5 to 19·0)	−20·9% (−26·6 to −14·2)
Italy	6 163 048 (5 684 428 to 6 714 537)	5156 (4792 to 5602)	−9·2% (−12·8 to −6·1)	14 292 (13 318 to 15 333)	7·3 (6·8 to 7·9)	−19·5% (−25·3 to −12·9)
Luxembourg	52 621 (48 612 to 57 232)	6011 (5558 to 6548)	−3·3% (−7·3 to 0·7)	85 (76 to 94)	7·7 (6·9 to 8·6)	−3·7% (−14·1 to 7·7)
Malta	45 196 (41 803 to 49 170)	6053 (5622 to 6550)	−5·3% (−8·6 to −1·7)	94 (88 to 102)	10·2 (9·5 to 11·0)	−20·7% (−27·6 to −13·6)
Netherlands	1 714 351 (1 585 981 to 1 860 677)	6142 (5688 to 6653)	1·3% (−3·1 to 6·1)	2683 (2511 to 2873)	7·1 (6·7 to 7·6)	6·9% (−0·5 to 15·8)
Norway	463 455 (430 874 to 500 803)	5767 (5363 to 6220)	10·1% (7·2 to 12·8)	590 (571 to 615)	5·3 (5·1 to 5·5)	34·5% (29·1 to 39·8)
Portugal	1 168 749 (1 079 819 to 1 269 003)	5817 (5416 to 6289)	−3·8% (−8·4 to 0·8)	3109 (2876 to 3361)	10·6 (9·8 to 11·4)	−7·6% (−14·7 to 0·3)
Spain	4 233 637 (3 900 640 to 4 624 353)	5034 (4647 to 5468)	−5·9% (−10·3 to −1·6)	10 605 (9890 to 11 361)	8·0 (7·5 to 8·6)	−32·3% (−37·5 to −26·5)
Sweden	1 128 448 (1 048 880 to 1 224 415)	6839 (6362 to 7400)	3·0% (0·3 to 5·7)	1461 (1370 to 1557)	5·7 (5·3 to 6·1)	57·5% (46·9 to 68·8)
Switzerland	841 113 (777 449 to 912 670)	5734 (5321 to 6199)	−1·2% (−5·7 to 3·5)	1558 (1449 to 1676)	7·1 (6·6 to 7·6)	103·9% (87·2 to 122·7)
UK	5 636 676 (5 233 735 to 6 135 943)	5167 (4819 to 5589)	−11·4% (−13·7 to −8·9)	6766 (6628 to 6903)	4·5 (4·4 to 4·6)	−14·5% (−16·3 to −12·6)
**Southern Latin America**	**5 750 645** (**5 380 566 to 6 189 240)**	**7402 (6928 to 7953)**	**3·9% (0·5 to 8·0)**	**15 847 (14 763 to 17 075)**	**18·8 (17·5 to 20·2)**	**−7·5% (−14·5 to 0·5)**
Argentina	3 841 009 (3 591 989 to 4 123 823)	7547 (7052 to 8109)	4·1% (0·0 to 8·7)	10 834 (9779 to 11 950)	19·7 (17·8 to 21·8)	−14·4% (−23·0 to −4·7)
Chile	1 569 089 (1 462 414 to 1 699 444)	7129 (6657 to 7716)	3·6% (0·3 to 7·3)	4225 (3829 to 4648)	18·1 (16·4 to 20·0)	26·5% (13·7 to 39·6)
Uruguay	340 293 (315 840 to 369 908)	7112 (6630 to 7681)	4·5% (−0·6 to 10·2)	787 (713 to 868)	12·9 (11·6 to 14·2)	−4·2% (−14·1 to 6·6)
**High-income North America**	**42 289 233** (**39 334 367 to 45 698 132)**	**7919 (7403 to 8540)**	**0·2% (−3·5 to 3·9)**	**91 038 (89 171 to 92 823)**	**13·8 (13·5 to 14·1)**	**57·3% (53·4 to 61·1)**
Canada	3 467 822 (3 213 111 to 3 766 495)	6023 (5615 to 6523)	5·9% (−0·2 to 12·5)	6087 (5681 to 6544)	7·9 (7·4 to 8·5)	4·6% (−3·5 to 13·6)
Greenland	3965 (3669 to 4317)	6282 (5836 to 6806)	−1·8% (−5·7 to 2·9)	4 (3 to 5)	8·3 (6·0 to 9·2)	12·2% (−10·9 to 28·7)
USA	38 816 706 (36 156 443 to 41 956 816)	8144 (7615 to 8783)	0·1% (−3·6 to 3·8)	84 944 (83 154 to 86 756)	14·6 (14·3 to 14·9)	63·0% (58·8 to 66·9)
**Caribbean**	**4 304 951** (**4 005 320 to 4 639 975)**	**8591 (8000 to 9263)**	**3·5% (0·1 to 7·6)**	**11 023 (10 366 to 11 697)**	**21·8 (20·5 to 23·1)**	**17·2% (9·1 to 25·1)**
Antigua and Barbuda	9302 (8632 to 10 029)	9201 (8552 to 9907)	1·0% (−2·5 to 4·9)	26 (24 to 28)	26·4 (24·3 to 28·4)	8·3% (−2·9 to 18·8)
The Bahamas	35 741 (33 248 to 38 503)	9207 (8588 to 9927)	2·2% (−1·6 to 6·7)	93 (85 to 101)	25·9 (23·7 to 28·1)	14·0% (3·1 to 26·3)
Barbados	38 943 (36 182 to 42 090)	9135 (8514 to 9833)	3·2% (−0·9 to 7·5)	90 (82 to 97)	18·9 (17·3 to 20·4)	5·1% (−4·7 to 15·5)
Belize	28 612 (26 651 to 30 832)	9372 (8720 to 10 097)	8·8% (5·3 to 12·8)	84 (80 to 89)	31·9 (30·1 to 33·8)	41·5% (27·5 to 56·9)
Bermuda	8824 (8193 to 9609)	8278 (7730 to 8958)	0·8% (−3·4 to 4·9)	16 (15 to 18)	12·9 (11·9 to 14·0)	−20·7% (−28·3 to −12·0)
Cuba	1 315 812 (1 215 120 to 1 429 705)	7887 (7314 to 8545)	1·9% (−3·8 to 7·9)	2340 (2115 to 2584)	12·6 (11·4 to 14·0)	32·1% (19·6 to 47·0)
Dominica	8350 (7753 to 8987)	9774 (9081 to 10 542)	5·1% (−0·2 to 10·5)	33 (31 to 36)	35·7 (33·2 to 38·5)	26·3% (15·4 to 38·3)
Dominican Republic	814 154 (756 845 to 880 779)	8394 (7804 to 9101)	7·9% (3·2 to 13·3)	2308 (1876 to 2665)	25·0 (20·2 to 28·9)	74·4% (33·9 to 105·5)
Grenada	13 726 (12 800 to 14 781)	9768 (9100 to 10 526)	4·5% (0·2 to 8·4)	51 (47 to 54)	32·6 (30·4 to 34·8)	−0·5% (−8·3 to 9·1)
Guyana	63 209 (58 819 to 68 364)	9689 (9003 to 10 469)	8·7% (4·0 to 14·0)	181 (159 to 204)	30·1 (26·7 to 33·9)	39·6% (22·5 to 57·3)
Haiti	728 043 (675 837 to 786 398)	8904 (8281 to 9634)	−1·9% (−5·8 to 2·5)	2083 (1735 to 2499)	31·5 (26·4 to 37·5)	−6·3% (−22·0 to 11·1)
Jamaica	286 464 (266 400 to 308 671)	9775 (9094 to 10 543)	10·6% (7·0 to 14·7)	810 (696 to 929)	27·4 (23·5 to 31·5)	19·4% (0·4 to 39·1)
Puerto Rico	544 395 (504 959 to 588 309)	9378 (8726 to 10 113)	3·2% (0·2 to 6·5)	1691 (1581 to 1810)	23·1 (21·5 to 24·7)	−12·8% (−19·5 to −5·6)
Saint Lucia	19 023 (17 676 to 20 445)	9292 (8647 to 9980)	−0·7% (−4·9 to 3·5)	55 (51 to 60)	26·8 (24·5 to 29·0)	−5·6% (−14·9 to 3·9)
Saint Vincent and the Grenadines	12 863 (11 964 to 13 872)	9775 (9106 to 10 535)	6·6% (2·9 to 10·7)	37 (34 to 39)	27·0 (25·1 to 29·1)	17·4% (6·8 to 28·6)
Suriname	55 379 (51 366 to 59 805)	9464 (8784 to 10 199)	7·8% (3·7 to 11·6)	209 (188 to 231)	37·7 (34·0 to 41·5)	37·8% (21·8 to 53·9)
Trinidad and Tobago	152 776 (141 560 to 165 562)	8937 (8305 to 9664)	3·3% (−2·2 to 8·8)	473 (398 to 559)	27·0 (22·7 to 31·9)	19·9% (0·0 to 42·5)
Virgin Islands	14 527 (13 404 to 15 773)	9250 (8597 to 10 011)	5·1% (1·1 to 9·7)	48 (41 to 54)	27·3 (23·5 to 30·6)	30·1% (9·9 to 49·9)
**Andean Latin America**	**4 202 601 (3 920 607 to 4 538 911)**	**7473 (6965 to 8086)**	**5·1% (1·4 to 9·7)**	**14 191 (13 149 to 15 259)**	**26·5 (24·5 to 28·4)**	**9·1% (−0·2 to 19·8)**
Bolivia	713 975 (665 086 to 772 378)	7663 (7124 to 8287)	0·9% (−3·4 to 4·8)	3165 (2631 to 3769)	39·7 (33·1 to 47·1)	3·7% (−15·9 to 25·8)
Ecuador	1 207 828 (1 123 429 to 1 301 635)	7875 (7333 to 8491)	11·3% (6·7 to 16·4)	5739 (5253 to 6242)	40·2 (36·8 to 43·8)	95·5% (78·0 to 114·0)
Peru	2 280 798 (2 123 558 to 2 472 702)	7221 (6710 to 7840)	3·3% (−1·9 to 9·1)	5287 (4609 to 6059)	16·9 (14·7 to 19·4)	−24·0% (−34·2 to −11·4)
**Central Latin America**	**26 908 399 (25 096 568 to 28 953 310)**	**11 116 (10 358 to 11 979)**	**7·2% (4·6 to 10·3)**	**96 362 (93 273 to 99 062)**	**42·1 (40·8 to 43·3)**	**60·9% (52·7 to 66·2)**
Colombia	5 150 794 (4 775 434 to 5 611 276)	9638 (8935 to 10 505)	1·1% (−4·7 to 6·4)	8502 (7723 to 9370)	15·7 (14·2 to 17·3)	−34·1% (−40·5 to −26·9)
Costa Rica	505 459 (468 301 to 546 223)	10 173 (9441 to 10 974)	5·7% (0·7 to 11·5)	1265 (1173 to 1378)	25·6 (23·7 to 27·9)	37·5% (25·8 to 50·9)
El Salvador	618 749 (575 374 to 669 028)	10 650 (9882 to 11 530)	12·4% (9·4 to 15·6)	4107 (2813 to 4846)	71·4 (48·7 to 84·2)	199·0% (46·7 to 270·8)
Guatemala	1 346 702 (1 253 013 to 1 449 862)	10 843 (10 059 to 11 704)	11·9% (7·9 to 16·4)	5065 (4616 to 5545)	47·8 (43·6 to 52·4)	37·5% (24·3 to 52·4)
Honduras	683 978 (635 326 to 738 666)	10 028 (9320 to 10 852)	10·4% (4·4 to 16·3)	744 (613 to 886)	13·0 (10·6 to 15·5)	29·5% (4·2 to 57·8)
Mexico	14 556 534 (13 572 422 to 15 614 239)	12 107 (11 292 to 13 009)	10·2% (7·7 to 13·0)	65 033 (63 122 to 66 615)	58·1 (56·4 to 59·5)	102·3% (94·4 to 108·8)
Nicaragua	559 208 (518 841 to 600 857)	10 989 (10 170 to 11 804)	2·4% (−1·0 to 6·1)	2292 (1959 to 2575)	49·4 (42·9 to 55·4)	20·9% (−6·3 to 39·5)
Panama	396 230 (367 921 to 428 061)	9979 (9264 to 10 775)	7·0% (2·0 to 12·4)	951 (889 to 1 019)	23·8 (22·2 to 25·5)	35·6% (24·5 to 47·4)
Venezuela	3 090 745 (2 860 732 to 3 350 686)	10 502 (9733 to 11 384)	3·5% (−0·4 to 7·7)	8403 (7418 to 9588)	31·3 (27·6 to 35·5)	54·9% (35·8 to 77·9)
**Tropical Latin America**	**17 263 386** (**16 035 424 to 18 629 836)**	**7365 (6842 to 7949)**	**0·1% (−2·5 to 3·1)**	**36 952 (36 137 to 37 742)**	**16·4 (16·1 to 16·8)**	**−0·2% (−2·7 to 2·2)**
Brazil	16 777 334 (15 579 858 to 18 107 349)	7337 (6817 to 7918)	−0·2% (−2·8 to 2·8)	35 350 (34 607 to 36 148)	16·1 (15·8 to 16·5)	−2·1% (−4·8 to 0·3)
Paraguay	486 053 (451 016 to 524 486)	8445 (7828 to 9144)	11·6% (6·7 to 17·0)	1602 (1287 to 1881)	31·2 (25·0 to 36·5)	87·3% (49·3 to 124·9)
**North Africa and Middle East**	**48 796 617** (**45 311 656 to 52 988 276)**	**10 361 (9616 to 11 247)**	**1·9% (−1·8 to 5·7)**	**74 269 (69 407 to 77 475)**	**19·7 (18·4 to 20·6)**	**−19·6% (−24·8 to −14·2)**
Afghanistan	1 586 966 (1 466 363 to 1 730 232)	10 913 (10 087 to 11 878)	−0·9% (−5·3 to 3·3)	3895 (3305 to 4605)	34·1 (29·4 to 39·9)	−24·5% (−38·5 to 15·2)
Algeria	3 497 207 (3 232 001 to 3 812 516)	9813 (9075 to 10 698)	0·9% (−3·5 to 5·8)	4577 (4074 to 5077)	15·6 (13·8 to 17·2)	−10·6% (−20·5 to 0·4)
Bahrain	132 052 (120 764 to 143 901)	10 427 (9660 to 11 291)	−1·7% (−6·6 to 3·5)	133 (118 to 148)	22·3 (19·7 to 24·6)	−42·6% (−49·3 to −34·6)
Egypt	7 101 539 (6 552 681 to 7 720 219)	10 572 (9754 to 11 521)	5·3% (1·4 to 9·6)	13 115 (11 314 to 14 968)	29·0 (25·0 to 33·1)	9·4% (−5·7 to 25·1)
Iran	8 339 849 (7 708 434 to 9 055 743)	10 924 (10 112 to 11 895)	11·3% (8·2 to 15·0)	10 163 (9381 to 10 549)	16·6 (15·4 to 17·3)	9·8% (−0·6 to 17·2)
Iraq	3 044 399 (2 826 656 to 3 295 432)	10 991 (10 186 to 11 946)	−9·3% (−13·0 to −5·7)	4706 (4245 to 5123)	21·9 (19·7 to 23·9)	−60·0% (−65·2 to −53·7)
Jordan	745 402 (691 588 to 808 791)	10 378 (9635 to 11 277)	−5·0% (−8·5 to −1·1)	1281 (1135 to 1451)	27·5 (24·5 to 31·0)	−18·0% (−30·3 to −3·4)
Kuwait	339 577 (313 180 to 369 112)	9716 (8988 to 10 559)	−0·2% (−6·0 to 6·3)	177 (160 to 201)	8·3 (7·5 to 9·5)	−64·1% (−67·7 to −59·6)
Lebanon	668 003 (618 911 to 726 949)	10 029 (9284 to 10 928)	−0·3% (−5·9 to 5·6)	594 (533 to 674)	11·2 (10·0 to 12·6)	−26·6% (−37·0 to −13·5)
Libya	594 010 (548 524 to 644 371)	10 963 (10 142 to 11 905)	6·6% (2·0 to 11·5)	1181 (1014 to 1363)	29·6 (25·6 to 33·9)	10·0% (−8·4 to 31·5)
Morocco	3 289 444 (3 046 873 to 3 568 865)	9924 (9201 to 10 750)	1·5% (−3·0 to 6·6)	4544 (3830 to 5334)	16·3 (13·9 to 19·1)	6·3% (−11·7 to 29·1)
Palestine	307 284 (283 987 to 333 626)	10 423 (9590 to 11 333)	−2·2% (−5·8 to 1·7)	624 (580 to 680)	28·5 (26·3 to 31·2)	−21·5% (−33·7 to −6·6)
Oman	324 395 (297 665 to 352 901)	10 611 (9836 to 11 554)	9·2% (4·2 to 14·9)	253 (198 to 298)	16·4 (12·4 to 19·2)	−3·5% (−23·1 to 20·0)
Qatar	188 200 (170 639 to 205 354)	9920 (9199 to 10 757)	−8·9% (−13·3 to −3·6)	115 (93 to 137)	26·5 (19·9 to 31·1)	−47·4% (−57·7 to −36·0)
Saudi Arabia	2 387 872 (2 201 128 to 2 595 630)	9892 (9139 to 10 812)	0·1% (−4·0 to 4·4)	3818 (3111 to 4411)	29·9 (24·2 to 34·1)	−2·6% (−21·7 to 22·7)
Sudan	2 274 010 (2 106 225 to 2 472 825)	10 107 (9337 to 11 001)	1·9% (−3·2 to 7·3)	3175 (2662 to 3765)	17·6 (14·7 to 20·9)	−22·6% (−36·1 to −7·7)
Syria	1 384 897 (1 271 191 to 1 505 808)	9881 (9099 to 10 748)	−4·8% (−8·8 to −0·1)	2257 (1951 to 2777)	19·7 (17·1 to 24·9)	−35·0% (−44·9 to −21·9)
Tunisia	1 218 223 (1 125 849 to 1 331 895)	9916 (9164 to 10 827)	5·1% (−0·6 to 11·9)	1645 (1358 to 1959)	15·3 (12·7 to 18·2)	−12·6% (−28·3 to 5·7)
Turkey	9 042 506 (8 350 852 to 9 874 836)	10 311 (9532 to 11 248)	−2·5% (−7·4 to 2·6)	15 153 (13 383 to 16 778)	17·8 (15·8 to 19·7)	−32·0% (−40·4 to −21·7)
United Arab Emirates	724 351 (655 751 to 795 089)	9951 (9198 to 10 828)	−4·0% (−7·7 to −0·1)	829 (648 to 1046)	30·8 (25·3 to 36·7)	3·0% (−18·1 to 29·0)
Yemen	1 560 862 (1 446 990 to 1 701 455)	9680 (8964 to 10 522)	1·4% (−3·0 to 6·3)	1964 (1541 to 2463)	16·4 (13·1 to 20·2)	−19·4% (−38·8 to 10·4)
**South Asia**	**143 173 973 (132 967 984 to 154 589 580)**	**9470 (8796 to 10 212)**	**6·3% (3·8 to 9·0)**	**282 464 (265 893 to 296 220)**	**22·8 (21·2 to 23·9)**	**−2·6% (−12·1 to 6·0)**
Bangladesh	11 196 657 (10 385 604 to 12 137 920)	8300 (7706 to 9014)	4·0% (−0·8 to 8·9)	16 783 (14 909 to 18 866)	15·4 (13·7 to 17·3)	−21·8% (−38·5 to −6·7)
Bhutan	69 290 (64 157 to 74 597)	9051 (8420 to 9756)	2·0% (−1·7 to 5·5)	136 (110 to 162)	23·3 (18·9 to 27·8)	−24·4% (−41·0 to −4·7)
India	115 069 914 (106 818 767 to 124 130 281)	9529 (8852 to 10 280)	5·6% (3·3 to 8·2)	223 821 (207 938 to 235 529)	22·3 (20·6 to 23·5)	−5·5% (−14·5 to 2·7)
Nepal	2 174 115 (2 019 410 to 2 351 912)	9136 (8487 to 9881)	7·9% (3·9 to 12·0)	4879 (3996 to 5789)	24·2 (20·0 to 28·6)	−4·0% (−21·6 to 16·0)
Pakistan	14 663 997 (13 591 223 to 15 834 072)	10 162 (9430 to 10 966)	14·3% (10·2 to 18·7)	36 844 (29 938 to 44 322)	33·9 (27·8 to 40·6)	42·3% (14·2 to 72·9)
**Central sub-Saharan Africa**	**6 969 028** (**6 467 179 to 7 541 973)**	**10 608 (9846 to 11 488)**	**0·0% (−3·6 to 3·7)**	**12 587 (11 086 to 14 024)**	**28·0 (24·9 to 31·0)**	**−17·6% (−28·2 to −5·6)**
Angola	1 499 658 (1 389 646 to 1 622 666)	10 592 (9813 to 11 468)	3·2% (−0·7 to 7·6)	2809 (2395 to 3225)	29·7 (25·7 to 34·1)	−19·2% (−33·0 to 0·1)
Central African Republic	285 293 (263 420 to 309 434)	10 648 (9868 to 11 533)	2·1% (−1·6 to 6·5)	655 (535 to 789)	32·9 (27·4 to 38·5)	−13·9% (−27·5 to 4·6)
Congo (Brazzaville)	347 706 (322 213 to 376 356)	10 938 (10 170 to 11 826)	−2·9% (−6·7 to 0·8)	671 (552 to 795)	31·4 (26·3 to 36·2)	−22·5% (−36·3 to −7·5)
Democratic Republic of the Congo	4 623 446 (4 286 990 to 5 002 633)	10 558 (9794 to 11 443)	−0·7% (−4·5 to 3·4)	8022 (6772 to 9290)	26·6 (22·6 to 30·5)	−17·3% (−31·6 to −0·3)
Equatorial Guinea	74 843 (69 512 to 80 983)	11 174 (10 350 to 12 064)	0·5% (−3·5 to 4·5)	134 (94 to 185)	32·4 (23·5 to 43·3)	−30·3% (−48·4 to −6·1)
Gabon	138 082 (128 236 to 149 087)	11 236 (10 438 to 12 148)	0·9% (−3·3 to 4·8)	296 (252 to 340)	32·4 (27·9 to 37·0)	−9·6% (−22·9 to 5·8)
**Eastern sub-Saharan Africa**	**20 233 557** (**18 793 079 to 21 887 248)**	**9764 (9054 to 10 549)**	**1·6% (−1·7 to 5·2)**	**37 332 (34 896 to 40 455)**	**25·9 (24·2 to 28·2)**	**−27·0% (−33·4 to −19·0)**
Burundi	535 340 (495 038 to 579 482)	9648 (8942 to 10 432)	−5·4% (−9·6 to −0·9)	972 (821 to 1147)	27·1 (23·3 to 31·4)	−35·0% (−45·8 to −23·2)
Comoros	51 396 (47 609 to 55 653)	9900 (9159 to 10 708)	−0·9% (−5·0 to 3·2)	104 (89 to 122)	26·3 (22·6 to 30·6)	−26·9% (−39·1 to −12·1)
Djibouti	73 613 (67 994 to 79 808)	9851 (9131 to 10 670)	6·6% (2·6 to 10·9)	139 (104 to 182)	30·8 (23·7 to 38·9)	0·2% (−22·4 to 31·3)
Eritrea	304 308 (282 168 to 329 461)	9868 (9168 to 10 679)	1·1% (−2·8 to 5·5)	641 (518 to 767)	30·4 (25·1 to 35·7)	−27·5% (−40·9 to −10·6)
Ethiopia	4 968 232 (4 617 353 to 5 364 731)	9376 (8694 to 10 139)	−3·9% (−7·1 to −0·4)	9083 (8190 to 10 165)	24·9 (22·5 to 28·1)	−50·5% (−58·0 to −40·7)
Kenya	2 682 885 (2 489 461 to 2 903 975)	9744 (9047 to 10 548)	6·5% (3·5 to 9·7)	4687 (4233 to 5204)	25·4 (22·8 to 28·5)	−2·7% (−9·4 to 4·4)
Madagascar	1 333 772 (1 230 299 to 1 444 892)	9632 (8917 to 10 446)	3·8% (−1·0 to 9·0)	2078 (1746 to 2424)	22·0 (18·8 to 25·6)	−19·2% (−31·9 to −4·3)
Malawi	982 874 (912 769 to 1 066 703)	10 371 (9610 to 11 246)	3·8% (−0·2 to 8·8)	1937 (1686 to 2188)	26·4 (22·9 to 29·9)	−15·8% (−32·0 to 17·9)
Mozambique	1 549 649 (1 434 294 to 1 673 132)	10 429 (9689 to 11 261)	4·5% (0·6 to 8·8)	2582 (2177 to 3312)	24·5 (20·6 to 32·2)	−10·0% (−24·8 to 6·6)
Rwanda	707 847 (654 743 to 769 522)	9771 (9043 to 10 599)	−1·4% (−6·3 to 3·7)	1148 (973 to 1345)	23·7 (20·3 to 27·6)	−43·0% (−52·2 to −32·5)
Somalia	842 140 (780 582 to 911 025)	10 019 (9278 to 10 833)	2·2% (−1·5 to 5·9)	2023 (1525 to 2640)	36·0 (28·1 to 45·2)	−13·7% (−34·8 to 20·5)
South Sudan	490 592 (454 696 to 530 633)	9731 (9023 to 10 540)	3·9% (0·4 to 8·0)	1241 (965 to 1604)	35·5 (28·0 to 44·8)	−11·7% (−33·8 to 21·2)
Tanzania	2 954 846 (2 740 477 to 3 216 811)	9737 (9009 to 10 607)	6·1% (1·4 to 10·9)	5806 (5091 to 6642)	25·3 (22·2 to 29·0)	−9·9% (−23·7 to 10·0)
Uganda	1 854 300 (1 717 949 to 2 010 440)	10 012 (9278 to 10 849)	1·1% (−3·3 to 5·6)	3015 (2581 to 3453)	24·4 (21·1 to 27·7)	−12·7% (−28·2 to 3·7)
Zambia	889 069 (824 745 to 959 071)	10 024 (9282 to 10 847)	4·7% (0·8 to 9·3)	1853 (1622 to 2099)	31·1 (27·1 to 35·1)	−25·1% (−37·0 to −11·7)
**Southern sub-Saharan Africa**	**7 255 813** (**6 732 619 to 7 830 539)**	**11 615 (10 808 to 12 512)**	**10·7% (8·0 to 13·5)**	**12 033 (11 337 to 12 625)**	**23·4 (22·1 to 24·6)**	**28·7% (17·9 to 38·0)**
Botswana	196 440 (182 104 to 211 968)	11 502 (10 689 to 12 412)	8·6% (4·9 to 12·6)	264 (232 to 306)	22·4 (19·7 to 25·8)	8·0% (−8·0 to 27·1)
eSwatini	88 896 (82 455 to 95 944)	12 568 (11 698 to 13 572)	8·8% (4·7 to 12·9)	213 (161 to 258)	39·2 (30·3 to 47·1)	16·9% (−7·1 to 43·9)
Lesotho	166 622 (154 774 to 180 465)	11 985 (11 143 to 12 979)	13·3% (9·2 to 17·5)	417 (338 to 501)	36·9 (30·2 to 43·7)	43·7% (15·7 to 75·1)
Namibia	177 623 (164 822 to 192 771)	10 740 (9961 to 11 654)	3·1% (−1·5 to 8·2)	250 (215 to 298)	18·9 (16·3 to 22·5)	−23·4% (−33·8 to −10·9)
South Africa	5 681 397 (5 270 490 to 6 129 994)	11 718 (10 895 to 12 637)	10·5% (7·8 to 13·2)	9149 (8584 to 9601)	22·3 (20·9 to 23·4)	28·8% (18·0 to 38·5)
Zimbabwe	944 836 (877 212 to 1 022 937)	11 055 (10 248 to 11 952)	12·3% (8·3 to 16·5)	1740 (1490 to 2054)	27·9 (24·1 to 32·6)	47·4% (23·9 to 78·6)
**Western sub-Saharan Africa**	**26 912 770** (**24 899 147 to 29 243 336)**	**11 326 (10 515 to 12 292)**	**4·3% (0·3 to 8·7)**	**39 718 (35 515 to 45 758)**	**20·6 (18·4 to 23·9)**	**−24·9% (−33·4 to −14·6)**
Benin	689 440 (640 678 to 745 414)	11 194 (10 368 to 12 149)	1·1% (−2·9 to 5·4)	1292 (1063 to 1553)	26·0 (21·7 to 31·4)	−21·8% (−35·1 to −6·7)
Burkina Faso	1 281 101 (1 185 130 to 1 383 412)	11 354 (10 524 to 12 288)	1·8% (−2·5 to 6·2)	2769 (2411 to 3147)	31·9 (27·8 to 36·2)	−7·9% (−21·1 to 9·4)
Cameroon	1 741 850 (1 622 811 to 1 889 103)	11 370 (10 591 to 12 359)	2·0% (−2·9 to 6·7)	3249 (2668 to 3 889)	27·9 (23·0 to 33·5)	−29·7% (−42·2 to −16·2)
Cape Verde	55 839 (51 817 to 60 741)	11 766 (10 921 to 12 780)	8·6% (4·0 to 13·1)	65 (59 to 72)	13·7 (12·5 to 15·1)	34·1% (12·0 to 54·0)
Chad	779 014 (723 905 to 844 703)	10 861 (10 088 to 11 771)	0·1% (−4·1 to 4·4)	1568 (1340 to 1852)	25·0 (21·2 to 30·0)	−14·8% (−27·5 to 0·6)
Côte d'Ivoire	1 642 666 (1 519 379 to 1 784 152)	11 613 (10 775 to 12 601)	−0·4% (−4·4 to 4·4)	2834 (2418 to 3404)	26·9 (23·1 to 32·4)	−19·6% (−33·2 to −3·7)
The Gambia	135 988 (126 031 to 147 163)	11 336 (10 507 to 12 316)	3·3% (−0·7 to 7·2)	243 (204 to 296)	25·1 (21·4 to 29·7)	−18·7% (−32·3 to −3·5)
Ghana	2 252 649 (2 090 611 to 2 441 403)	11 483 (10 673 to 12 422)	7·1% (2·6 to 12·0)	3572 (3055 to 4086)	21·9 (19·0 to 24·9)	16·7% (−4·2 to 38·0)
Guinea	747 095 (693 302 to 807 476)	11 218 (10 384 to 12 173)	1·6% (−2·7 to 6·2)	1598 (1392 to 1833)	28·1 (24·4 to 32·5)	−16·0% (−28·0 to −1·5)
Guinea-Bissau	116 129 (107 475 to 126 088)	11 989 (11 122 to 12 993)	0·8% (−3·3 to 5·5)	260 (216 to 315)	34·3 (29·0 to 40·8)	−33·0% (−44·8 to −19·5)
Liberia	308 302 (285 351 to 333 730)	11 511 (10 683 to 12 463)	0·7% (−3·4 to 5·3)	517 (429 to 627)	25·8 (21·4 to 31·3)	−29·5% (−40·8 to −15·7)
Mali	1 127 953 (1 044 615 to 1 219 025)	10 783 (9978 to 11 711)	−2·5% (−6·6 to 1·6)	2144 (1819 to 2546)	22·7 (19·3 to 27·2)	−36·1% (−45·8 to −24·7)
Mauritania	271 447 (251 721 to 294 100)	11 281 (10 440 to 12 231)	−4·2% (−7·9 to 0·1)	460 (385 to 549)	23·8 (20·0 to 28·4)	−38·8% (−49·5 to −26·8)
Niger	1 047 090 (970 398 to 1 130 392)	10 774 (9978 to 11 713)	−1·7% (−5·8 to 2·9)	1777 (1433 to 2203)	21·8 (17·7 to 27·3)	−35·4% (−46·5 to −21·8)
Nigeria	12 681 837 (11 675 878 to 13 853 971)	11 387 (10 498 to 12 386)	7·8% (1·9 to 14·0)	13 740 (10 420 to 18 751)	15·0 (11·3 to 20·6)	−34·1% (−50·0 to −11·3)
São Tomé and Príncipe	15 734 (14 575 to 17 056)	12 188 (11 288 to 13 210)	0·8% (−3·3 to 5·2)	45 (39 to 52)	44·1 (38·1 to 50·5)	15·9% (−2·5 to 35·8)
Senegal	996 298 (924 932 to 1 079 051)	11 236 (10 433 to 12 176)	−0·6% (−4·6 to 4·0)	1930 (1668 to 2282)	27·0 (23·2 to 32·0)	−27·2% (−37·6 to −14·9)
Sierra Leone	510 314 (472 593 to 552 280)	11 412 (10 580 to 12 389)	2·0% (−1·7 to 6·4)	896 (775 to 1050)	24·1 (20·9 to 28·2)	−23·7% (−36·8 to −5·9)
Togo	511 758 (472 213 to 554 594)	11 396 (10 547 to 12 341)	2·3% (−2·0 to 7·2)	759 (629 to 907)	22·4 (18·9 to 26·2)	−24·5% (−37·6 to −10·2)

UI=uncertainty interval. SDI=Socio-demographic Index.

In 2017, the prevalence of CKD was estimated as 9·1% (95% UI 8·5 to 9·8) in the world's population, with CKD stages 1–2 accounting for 5·0% (4·5 to 5·5), stage 3 for 3·9% (3·5 to 4·3), stage 4 for 0·16% (0·13 to 0·19), stage 5 for 0·07% (0·06 to 0·08), dialysis for 0·041% (0·037 to 0·044), and kidney transplantation for 0·011% (0·010 to 0·012). The age-standardised prevalence of CKD was 1·29 (95% UI 1·28 to 1·30) times higher in females (9·5% [8·8 to 10·2]) than in males (7·3% [6·8 to 7·9]). The global age-standardised incidence of dialysis and transplantation was 1·47 (95% UI 1·46 to 1·48) times greater among males (13·7 [12·6 to 14·9] per 100 000 population) than among females (8·6 [7·9 to 9·3] per 100 000 population). The global age-standardised CKD mortality rate was 1·39 (1·30 to 1·45) times greater among males (18·9 [17·9 to 19·5] per 100 000 population) than among females (13·6 [13·3 to 14·0] per 100 000 population). The country median prevalence of CKD was 8·9% (IQR 7·1 to 10·8).

The global age-standardised prevalence of CKD has remained stable between 1990 and 2017, with a 1·2% (95% UI −1·1 to 3·5) change, although the all-age prevalence of CKD has increased by 29·3% (26·4 to 32·6) from 1990 to 2017. The availability of renal replacement therapy from 1990 to 2017 has grown; global all-age incidence of dialysis and kidney transplantation increased by 43·1% (95% UI 40·5 to 45·8) and 34·4% (29·7 to 38·9), respectively, while global age-standardised incidence rose by 10·7% (9·1 to 12·3) and 12·8% (10·2 to 15·3), respectively, with larger increases in all-age incidence of renal replacement therapy resulting from population ageing. Despite such increases, availability of renal replacement therapy is still limited in many world regions with a high burden of CKD.

### Global mortality

CKD resulted in 1·2 million (95% UI 1·2 to 1·3) deaths in 2017 (table 2). An additional 1·4 million (1·2 to 1·6) deaths from cardiovascular disease were attributable to impaired kidney function, representing 7·6% (6·5 to 8·8) of deaths due to cardiovascular disease in 2017. Deaths due to CKD, and cardiovascular disease deaths attributable to impaired kidney function, represented 4·6% (95% UI 4·3 to 5·0) of total mortality. Ranked as the 17th leading cause of death in 1990, CKD has increased in importance, ranking as the 12th leading cause of death in 2017. The rank of CKD among all causes of death was especially prominent in Central and Andean Latin America, ranking second and fifth, respectively. The global all-age CKD mortality rate increased by 41·5% (95% UI 35·2 to 46·5) from 1990 to 2017. The age-standardised mortality rate remained stable, with a 2·8% change (−1·5 to 6·3) from 1990 to 2017. The age-standardised CKD mortality rate, however, has increased by 60·9% (95% UI 52·7 to 66·2) in central Latin America, 60·9% (53·3 to 68·9) in central Asia, and 57·3% (53·4 to 61·1) in high-income North America.

### YLDs, YLLs, and DALYs

**Figure 1:**
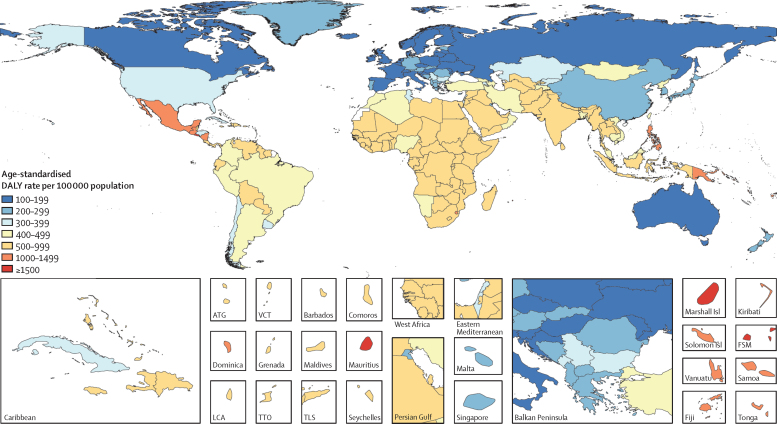
Age-standardised rate of DALYs for chronic kidney disease in 2017 DALY=disability-adjusted life-year. ATG=Antigua and Barbuda. FSM=Federated States of Micronesia. LCA=Saint Lucia. TLS=Timor-Leste. TTO=Trinidad and Tobago. VCT=Saint Vincent and the Grenadines.

In 2017, CKD resulted in 7·3 million (95% UI 5·4 to 9·2) YLDs, 28·5 million (27·6–29·3) YLLs, and 35·8 million (33·7 to 38·0) DALYs (appendix pp 16–26). Although most prevalent cases of CKD are stages 1–2 or stage 3, YLDs are largest for CKD stage 5 and dialysis, which accounted for 40% and 22% of YLDs for CKD, respectively, in 2017. Considerable global variation was noted in CKD burden, with age-standardised CKD DALY rates varying more than 15-fold between countries (figure 1). American Samoa, El Salvador, Federated States of Micronesia, Marshall Islands, and Mauritius had the highest estimated rates of age-standardised CKD DALYs in 2017, with more than 1500 DALYs per 100 000 population, whereas Andorra, Finland, Iceland, and Slovenia had the lowest burden, with age-standardised DALYs for CKD of less than 120 per 100 000 population. Despite the relatively high prevalence of non-fatal CKD, YLDs accounted for only 20·3% (95% UI 15·9 to 24·6) of total CKD DALYs in 2017, because most of the prevalence of CKD was contributed by disease stages 1–2 and stage 3, which confer little to no functional health loss. While slight increases were estimated in CKD prevalence and mortality from 1990 to 2017, global age-standardised rates of YLLs and YLDs due to CKD decreased over this period by 9·6% (95% UI 5·8 to 13·1) and 4·3% (0·4 to 9·0), respectively, thereby resulting in an 8·6% (5·4 to 11·8) decrease in the global age-standardised rate of DALYs due to CKD.

### Burden by SDI

**Figure 2:**
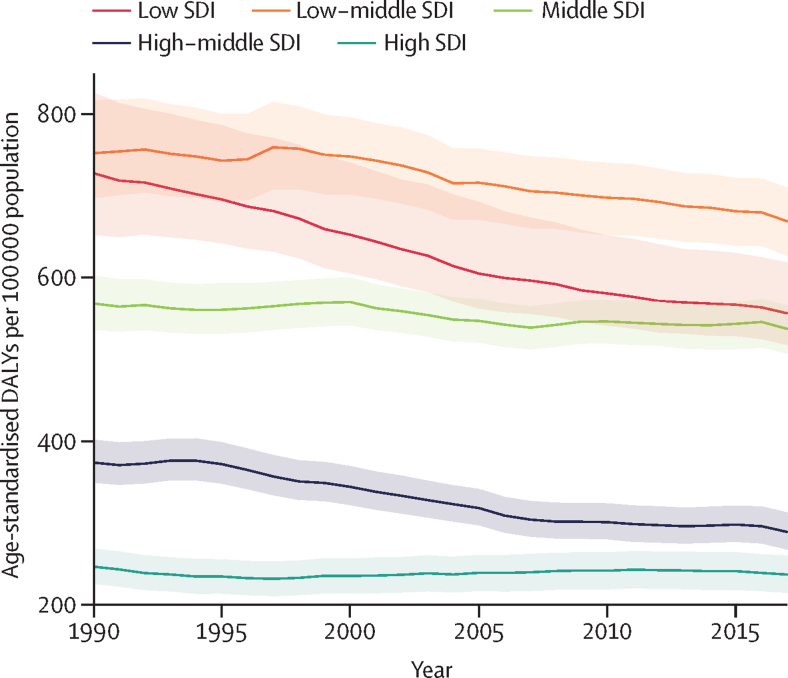
Age-standardised rate of DALYs by SDI quintiles for chronic kidney disease, 1990–2017 Shaded areas represent 95% uncertainty intervals. DALY=disability-adjusted life-year. SDI=Socio-demographic Index.

The age-standardised DALY rate due to CKD was highest in the low-middle SDI quintile, and was lower in the high and high-middle SDI quintiles than in the other quintiles in 2017 (appendix pp 26–31). The low SDI quintile saw the largest change in age-standardised DALY rates, changing by −23·6% (95% UI −29·2 to −16·2) between 1990 and 2017. The high and middle SDI quintiles saw the smallest changes, with age-standardised DALY rates changing by −4·0% (−6·5 to −1·8) and −5·5% (−9·3 to −2·3), respectively, since 1990 (figure 2). Despite declining age-standardised DALY rates, all SDI quintiles showed a net increase in the absolute number of DALYs attributable to CKD from 1990 to 2017, attributable to population growth and ageing.

**Figure 3:**
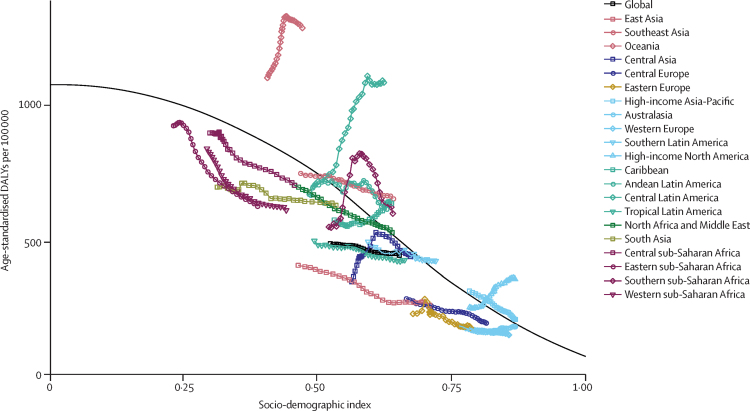
Age-standardised rate of DALYs for chronic kidney disease for 21 world regions, 1990–2017 Solid black line shows expected values across the spectrum of the SDI. DALY=disability-adjusted life-year. SDI=Socio-demographic Index.

The estimated relation between SDI and expected age-standardised rate of CKD DALYs is generally negative, with the slope steepening as SDI increases (figure 3). Oceania had much higher age-standardised DALY rates due to CKD than expected based on SDI for all years between 1990 and 2017. Despite gains in SDI over time, central Latin America, high-income North America, and the Caribbean also saw increasing rates of CKD DALYs, by contrast with other world regions. Western, eastern, and central sub-Saharan Africa, east Asia, south Asia, central and eastern Europe, Australasia, and western Europe all had a lower age-standardised CKD DALY rate than expected, based on their SDI values.

### Causal attribution

**Figure 4:**
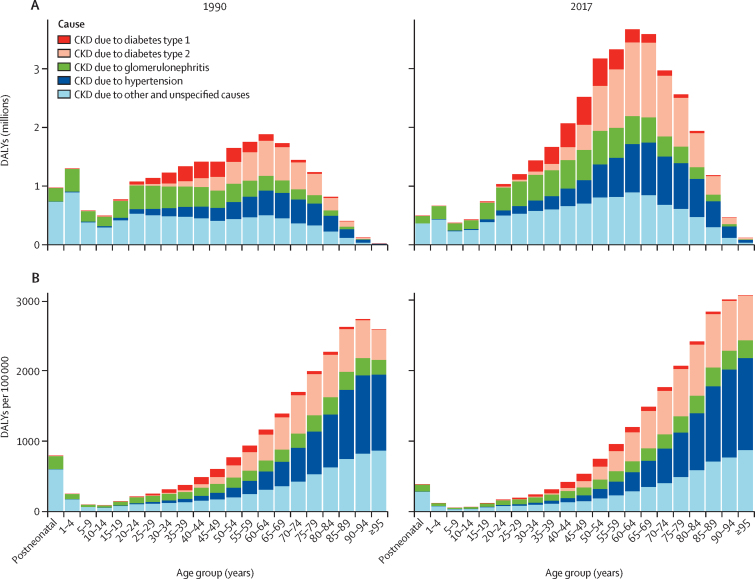
Number (A) and rate (B) of global DALYs for CKD by underlying cause in 1990 and 2017 DALY=disability-adjusted life-year. CKD=chronic kidney disease.

CKD due to diabetes accounted for 30·7% (95% UI 27·8 to 34·0) of CKD DALYs, the largest contribution in terms of absolute number of DALYs of any cause in 2017, with CKD due to type 1 and type 2 diabetes resulting in 2·9 million (2·4 to 3·5) DALYs and 8·1 million (7·1 to 9·2) DALYs, respectively (figure 4A). CKD due to other and unspecified causes resulted in a substantial number of DALYs across all age groups and was estimated to have the highest age-standardised rate of DALYs (138·8 [95% UI 123·7 to 155·9] per 100 000 population) among all causes of CKD. Type 2 diabetes was the only cause of CKD to show a significant increase in the age-standardised DALY rate, which changed by 9·5% (95% UI 4·3 to 13·7) from 1990 to 2017 (figure 4B).

### Risk factors for CKD

Impaired fasting plasma glucose, high blood pressure, high body-mass index, a diet high in sodium, and lead were risk factors for CKD quantified in GBD, accounting for 57·6% (95% UI 50·5 to 63·8), 43·2% (42·3 to 54·1), 26·6% (17·0 to 37·7), 9·5% (3·7 to 18·0), and 3·6% (2·3 to 5·1), respectively, of the age-standardised rate of CKD DALYs in 2017. High blood pressure accounted for the largest proportion of CKD burden in east Asia, eastern Europe, tropical Latin America, and western sub-Saharan Africa, whereas high fasting plasma glucose was the leading risk factor for CKD in all other regions.

### Impaired kidney function as a risk factor

**Figure 5:**
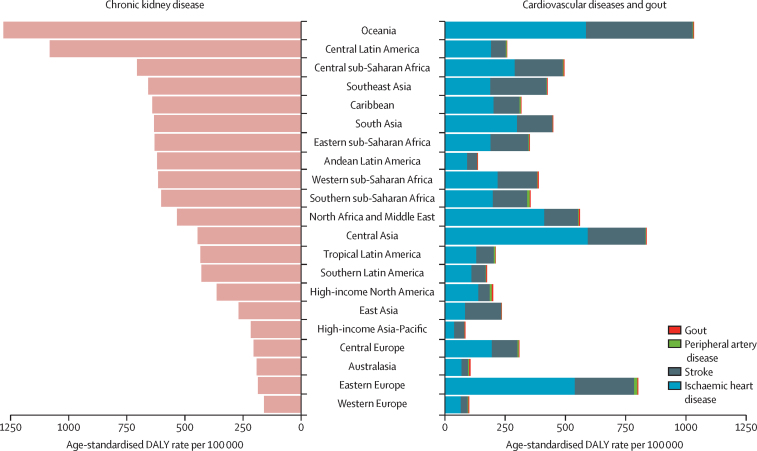
Age-standardised rate of DALYs for chronic kidney disease, cardiovascular diseases, and gout, attributable to impaired kidney function, for 21 world regions in 2017 DALY=disability-adjusted life-year.

In 2017, impaired kidney function resulted in 61·3 million (95% UI 56·9 to 66·1) DALYs, of which 58·4% (55·4 to 61·4) were directly contributed by CKD whereas 41·6% (38·2 to 44·4) were cardiovascular disease DALYs and less than 1% (0·003% [95% UI 0·002 to 0·004]) were gout DALYs attributable to impaired kidney function, although the composition was different by world region (figure 5). Of 25·3 million (95% UI 22·2 to 28·9) DALYs resulting from cardiovascular disease attributable to impaired kidney function, 58·8% (54·0 to 63·7) came from ischaemic heart disease, 40·2% (35·4 to 45·0) from stroke, and the remaining 1·0% (0·6 to 1·5) from peripheral artery disease. The global age-standardised rate of cardiovascular disease DALYs attributable to impaired kidney function in 2017 was 318·3 (278·9 to 363·2) DALYs per 100 000 population. With an age-standardised rate of 1028·4 (866·0 to 1218·8) DALYs per 100 000 population, Oceania had the largest age-standardised rate of cardiovascular disease DALYs attributable to impaired kidney function, followed by central Asia, eastern Europe, and north Africa and the Middle East, which all had age-standardised DALY rates above 500 per 100 000 population. Since 1990, the age-standardised rate of cardiovascular disease DALYs due to impaired kidney function has decreased globally by 29·4% (95% UI 26·4 to 32·2), although central Asia saw a rise of 10·5% (4·9 to 16·3).

Information on input data sources for cause of death, non-fatal, and risk factor models can be downloaded from the Global Health Data Exchange.^60^ Complete GBD 2017 results are available online for visualisation and can be downloaded from the Global Health Data Exchange.^61^

## Discussion

The number of individuals with all-stage CKD reached almost 700 million in 2017, which is more people than those with diabetes, osteoarthritis, chronic obstructive pulmonary disease (COPD), asthma, or depressive disorders.^26^ CKD diagnoses resulted in 1·2 million deaths in 2017, a number that has been projected to rise by 2040 to 2·2 million in a best-case scenario and up to 4·0 million in a worst-case scenario.^62^ GBD ranks CKD as the 12th leading cause of death out of 133 conditions.^28^ Globally in 2017, CKD resulted in more deaths than did tuberculosis or HIV, and the number of CKD deaths was almost equal to the number of deaths due to road injuries.^27^ The decrease in the global age-standardised DALY rate for CKD from 1990 to 2017 was not accompanied by corresponding decreases in age-standardised rates of CKD mortality or prevalence, indicating a shift to mortality due to CKD occurring at an older age and lower average severity of non-fatal CKD. However, these declines are slower than for cardiovascular disease and stroke. A handful of locations deviated from this global trend and had a greater than 50% increase in the age-standardised CKD DALY rate from 1990 to 2017, including American Samoa, Ecuador, El Salvador, Georgia, Guam, Mexico, Paraguay, and Philippines. The increases in CKD burden in these locations have been largely driven by substantial increases in the CKD mortality rate. Although there is still uncertainty about the cause of CKD in some populations with high CKD mortality,^63^ studies from American Samoa,^64^, ^65^ Guam,^64^ and Mexico^66^, ^67^ suggest that limited access to renal replacement therapy combined with increases in the prevalence of diabetes and hypertension have contributed to rising CKD burden in these regions. We found a higher prevalence of CKD stages 1–3 in females and higher mortality in males, which suggests that males progress to ESKD more rapidly. There is evidence of substantial gender disparities in access to CKD treatment, and actions are needed to provide equal access to kidney health care.^68^, ^69^ These results highlight the importance of improved management of risk factors for CKD at primary care level and the need for expanded access to affordable renal replacement services for those with ESKD.

We have not seen the same degree of progress in prevention of CKD mortality as we have for many other important non-communicable diseases. From 1990 to 2017, the global age-standardised mortality rate declined by 30·4% for cardiovascular disease, 14·9% for cancer, and 41·3% for COPD,^27^ but a similar decline was not seen for CKD (2·8% change [95% UI −1·5 to 6·3]). Disparities in CKD mortality by world region highlight the importance of access to renal replacement therapy, both to initiate treatment and to maintain access to dialysis. For instance, in sub-Saharan Africa, even if individuals requiring renal replacement initiate treatment, retention is low because of an inability to pay for ongoing dialysis, and up to 85% of incident patients are forced to withdraw from this life-saving treatment.^70^ Public health policy also has a role in slowing the incidence rate of ESKD through education of health personnel, early kidney disease detection programmes and implementation of nephroprotective treatment, and appropriate treatment of CKD risk factors such as high systolic blood pressure and elevated glucose levels.^15^ This action could be especially important in low SDI regions where primary care health systems are focused on child and maternal health and are less equipped to adequately prevent and treat chronic diseases.^71^ In view of the detrimental interplay between diabetes, hypertension, CKD, and development of cardiovascular disease, one alternative could be to integrate screening of CKD in ongoing efforts to reduce the occurrence of cardiovascular disease. Studies suggest that screening for CKD in high-risk and elderly populations is a cost-effective approach to reduce progression to ESKD and CKD mortality.^72^, ^73^, ^74^, ^75^ The effect of kidney disease on the burden of non-communicable diseases is not limited to ESKD or CKD itself but extends to cardiovascular diseases for which impaired kidney function is an independent risk factor.^19^, ^22^ According to our analysis, almost 7% of the total cardiovascular disease burden can be attributed to impaired kidney function. Of all the global risk factors in 2017, the DALY rate for impaired kidney function was higher than that for drug use, unsafe sanitation, low physical activity, second-hand smoke, and several dietary risk factors.^29^ However, kidney health is given far less attention than the aforementioned risk factors, both by public health authorities and the general population. Fewer than 10% of patients with CKD are aware of their disease, both in developing^14^ and developed^76^ countries. According to the Global Kidney Health Atlas 2017, CKD is recognised as a health-care priority in 36% of countries, a national strategy for combating all stages of CKD is available in 17% of countries, and awareness and adoption of CKD guidelines among primary and secondary care specialists is estimated to be below average in 49% of countries.

Kidney care is closely related to global health challenges. The gap between age-standardised CKD DALY rates in low, low-middle, and middle SDI quintiles compared with high and high-middle SDI quintiles reflects inequities in access to preventive care and renal replacement therapy across levels of development. The low SDI quintile saw the largest decrease in age-standardised CKD DALY rates from 1990 to 2017, primarily as a result of substantial decreases in the age-standardised mortality rate in many countries in the low SDI quintile, including Ethiopia, Niger, and Rwanda. Nevertheless, the fraction of all deaths in these countries attributable to CKD has steadily increased over the past 20 years, indicating that differences in CKD burden observed between low-middle and low SDI quintiles might diminish when overall declines in all-cause mortality rate slow, particularly as exposures to risk factors for CKD, including diabetes, hypertension, and high body-mass index, continue to amplify in the low SDI quintile. For these countries at the lower end of the development spectrum, the situation has been aggravated by insufficient access to laboratory diagnostic services,^77^ a shortage of medical personnel,^78^ scarce medication, and the absence of universal health coverage.^79^ Disparities in access to CKD care also exist within countries for different populations, some of which are at higher risk of late referral to nephrology services,^80^ resulting in greater rates of complications and mortality.^81^

The prevalence of CKD reported in GBD 2017 (9·1% [95% UI 8·5 to 9·8]) is lower than the global prevalence estimate from a meta-analysis published in 2016 (13·4%).^82^ This difference is attributable to variations in methodological approach and data inclusion criteria. For the estimation of CKD prevalence, GBD excluded studies reporting on clinic-based or laboratory-based populations, because patients with creatinine samples for CKD in these instances tend to belong to high-risk groups, leading to overestimation of CKD prevalence. Moreover, GBD also accounted for differences in age and sex distributions across input data sources, thus ensuring that data affected prevalence estimates only for the demographic subset from which they were gathered. Estimates of CKD prevalence in GBD 2017 are higher than those reported in previous iterations of GBD, primarily because of inclusion of CKD stages 1–2 as part of the GBD definition of CKD for GBD 2017.

Several limitations applicable to GBD have been discussed in detail elsewhere.^26^, ^27^, ^28^, ^29^ Limitations specific to CKD apply to the current analysis.

First, many countries do not have high-quality population-based studies on the occurrence of CKD. Some regions with the highest burden of CKD (eg, Central America, Latin America, and Oceania) have little to no available data for CKD incidence or prevalence within the population (appendix p 7); as such, GBD must rely on statistical methods and predictive covariate values to estimate CKD burden in these regions. Additionally, despite the standardised definition of CKD presented by the KDIGO guidelines,^22^ sources of information on non-fatal CKD vary in terms of sampling, laboratory methods, and the equation used to calculate eGFR. Several studies have noted systematic differences in estimated CKD prevalence resulting from the use of different equations.^46^, ^83^, ^84^, ^85^, ^86^, ^87^, ^88^ To account for these differences and standardise data across studies, we adjusted data sources to represent prevalence as calculated according to our reference definition of eGFR, estimated using the CKD-EPI equation.^32^ This correction increases the uncertainty of input data and was derived from a limited number of sources, which might not accurately characterise bias within different demographic subsets of the population. Future iterations of GBD should expand the number of sources used for this analysis to evaluate if these adjustment factors vary by sex, age, ethnicity, or geographical location.

Second, most data sources reporting the prevalence of non-fatal CKD are cross-sectional and do not repeat serum creatinine and urine ACR measurements over 3 months, as suggested by KDIGO guidelines, to confirm the chronicity of abnormalities.^22^ Studies suggest that use of one measurement of decreased eGFR to characterise CKD might overestimate prevalence by 25–50%.^89^, ^90^, ^91^, ^92^ Therefore, it is possible that the results of our analysis represent an overestimate of CKD prevalence. Future analyses of the global burden of CKD should investigate developing a methodology to correct prevalence estimates based on one eGFR measurement, as done to correct for differences in prevalence estimates resulting from use of alternate estimating equations.

Third, ascertainment of the cause of CKD is difficult. Biopsy is the gold-standard method for assigning the underlying cause of CKD, but this procedure is only advised when confirmation of cause is necessary and the benefits of confirmation outweigh risks of the procedure. Additionally, CKD often arises from comorbid conditions, contributing to a large degree of uncertainty about the true underlying cause of CKD. To capture this uncertainty and better reflect clinical realities of causal attribution, we used data from the Geisinger Health System^35^ to identify patients with various stages of CKD, relying on ICD codes for primary renal diseases to map individuals to GBD cause groupings and assigning individuals with CKD but no ICD code for a primary renal disease as having CKD of unknown cause. This method marks an improvement over previous iterations of GBD in which the distribution of CKD by cause was generated using data solely from renal replacement therapy registries and was assumed to be the same across all stages. However, the generalisability of Geisinger data to CKD populations worldwide is limited, because there is considerable geographical variation in the distribution of primary renal diseases. Use of clinical data also probably represents a skewed causal distribution in early-stage CKD, because many cases of mild kidney function decline are asymptomatic, so there could be differential case-ascertainment based on primary renal disease.

Fourth, impaired kidney function as defined for GBD risk factor estimation does not explicitly quantify the risk of elevated ACR and decreased eGFR separately, despite evidence that elevated ACR leads to increased risk for cardiovascular outcomes independent of decreased eGFR.^93^ Estimation of attributable burden separately by ACR and eGFR would provide additional insights for treatment and prevention of cardiovascular disease outcomes among individuals with impaired kidney function. We also only quantified the burden of cardiovascular disease and gout attributable to impaired kidney function; however, impaired kidney function might also put individuals at risk for other outcomes that we have not yet accounted for. Additional outcomes for impaired kidney function could be added to future GBD iterations if evidence can establish a causal link.

Finally, the GBD estimation framework for CKD does not currently estimate anaemia impairment for cases of ESKD on dialysis or ESKD after transplant. Patients on dialysis have a high prevalence of anaemia, the severity of which could affect the level of disability they experience. The disability weight associated with dialysis is relatively high; however, the lay description associated with this health state also includes symptoms associated with anaemia. Nevertheless, this might have resulted in an underestimate of the disability associated with dialysis.

In conclusion, CKD is a highly prevalent condition that contributes a substantial proportion of disease burden globally, yet over the past 27 years the burden of CKD has not declined to the same extent as many other important non-communicable diseases. The age-standardised effect of CKD is more prominent in countries in low and middle SDI quintiles, where there is a large gap between CKD burden and provision of adequate health care. Importantly, slowing CKD progression at early stages provides economic benefits^94^ and prevents the development of ESKD and cardiovascular complications.^22^ A comprehensive action plan should include effective management of risk factors for CKD at the primary care level, improved case-detection among at-risk populations, and development of facilities for treating patients with documented disease. By jointly implementing CKD prevention, assessment, and treatment, we could achieve better health for many populations with a high burden of CKD and related outcomes. The UN Sustainable Development Goals aim to reduce premature mortality from non-communicable diseases by a third by 2030. Our estimates suggest that targeting CKD could be an important step in reaching these goals.
