# Supplementary material for: Global, regional, and national burden of chronic kidney disease, 1990–2017: a systematic analysis for the Global Burden of Disease Study 2017
Source: Lancet. 2020 Feb 29;395(10225):709–33. doi: 10.1016/S0140-6736(20)30045-3 (PMC7049905; doi:10.1016/S0140-6736(20)30045-3)
Supplement: Supplementary appendix [file mmc1.pdf]

THE LANCET

Supplementary appendix

This appendix formed part of the original submission and has been peer reviewed. We post it as supplied by the authors.

Supplement to: GBD Chronic Kidney Disease Collaboration. Global, regional, and national burden of chronic kidney disease, 1990–2017: a systematic analysis for the Global Burden of Disease Study 2017. *Lancet* 2020; published online Feb 13. http://dx.doi.org/10.1016/S0140-6736(20)30045-3.

Contents

Methods Appendix 2
Mortality estimation 2
Input data 2
Modelling strategy 2
Non-fatal estimation 6
Case definition 6
Input data 6
Modelling strategy 9
Risk factor estimation 14
Modelling strategy 14
Theoretical minimum risk exposure level 14
Outcomes 14
Relative risk 14
Population attributable fraction 14
Supplemental results 15
Burden by region 15
Supplemental tables 16
GATHER checklist 32

Methods Appendix

Mortality estimation

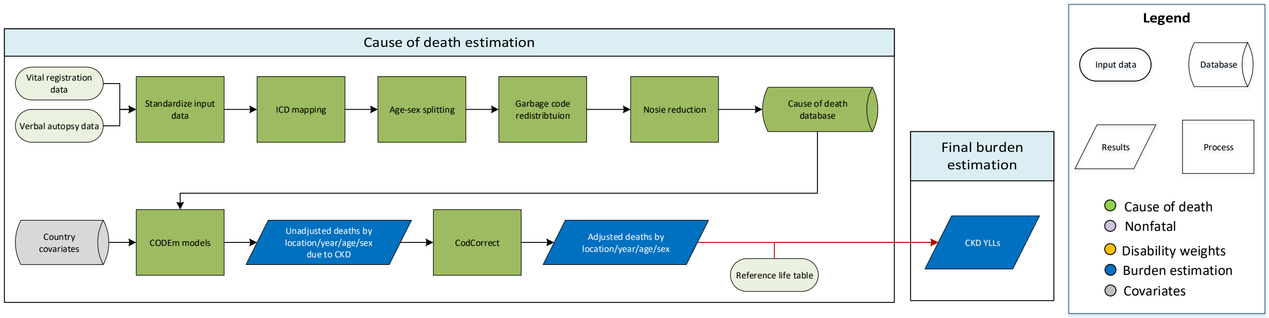

Input data

Vital registration and verbal autopsy data were used to model mortality due to chronic kidney disease. Data were standardised and mapped according to the GBD causes of death ICD mapping method, which assigns each death to a single underlying cause. ICD 9 codes mapped to CKD include 250.4, 403-404.9, 581-583.9, 585-585.9, 589-589.9, 753-753.3. ICD 10 codes mapped to CKD include D63.1, E10.2, E11.2, E12.2, E13.2, E14.2, I12-I13.9, N02-N08.8, N15.0, N18-N18.9, Q61-Q62.8. Deaths due to congenital kidney anomalies (cystic kidney disease and reflux hydronephrosis) were attributed to CKD, making a change from previous iterations of GBD when these deaths were attributed to urogenital congenital anomalies.

These data were then age-sex split, and appropriate redistribution of garbage code data was performed. Nonspecific codes, such as senility or unspecified causes, were redistributed proportionally across all GBD causes. Other codes not corresponding to a most detailed GBD cause were assigned to the appropriate level within the GBD cause hierarchy and redistributed to all causes within that level. For example, unspecified endocrine disorders was redistributed to every cause within the Level 2 group diabetes, urogenital, blood, and endocrine diseases.

Outliers were identified by systematic examination of data points for all location-years. Data points that violated well-established age or time trends or that resulted in extremely high or low cause fractions (i.e. the ratio of the count of deaths for a specific cause to the total number of deaths in an age, year, location and year category) were determined to be outliers.

Modelling strategy

A standard CODEm model with location-level was used to model deaths due to chronic kidney disease. Iterations of models were assessed at the location/year/age-group/sex level to determine whether data points merited exclusion via outliering. Unadjusted death estimates were adjusted using CoDCorrect to produce final estimates of YLLs. The covariates used are displayed below.

Table S1 - CKD CODEm covariates

Level	Covariate	Direction
1	Diabetes fasting plasma glucose (mmol/L)	+
	Diabetes age-standardised prevalence (proportion)	+
	Mean systolic blood pressure (mmHg)	+
	Mean BMI	+
	Healthcare Access and Quality index	−
2	Mean cholesterol	+
	Total calories (kcal per capita)	−
	Red meat (kcal per capita)	0
3	Socio-demographic Index	0
	Education (years per capita)	−
	Log LDI ($I per capita)	−

Separate models were run for males and females and for a global model (including all countries and subnational locations included in GBD) and a 'data-rich' model (including countries and subnational lcoations that meet a standard of high coverage and quality (by means of assessing the proportion of specific codes as opposed to less informative 'garbage' codes) of vital registration data from 1980 until the most recent year of estimation. In the data rich model we apply less smoothing over time and age to allow the model to better track the data. The data-rich models also create less uncertainty than the global models which include many countries with lesser quality data.

The following plots show the covariate influence in the male and female, global and data-rich models. The plots show the standardised beta values for each covariate averaged across all 1,000 draws. The size of the bar reflects how influential each covariate has been in the model.

Female, global model

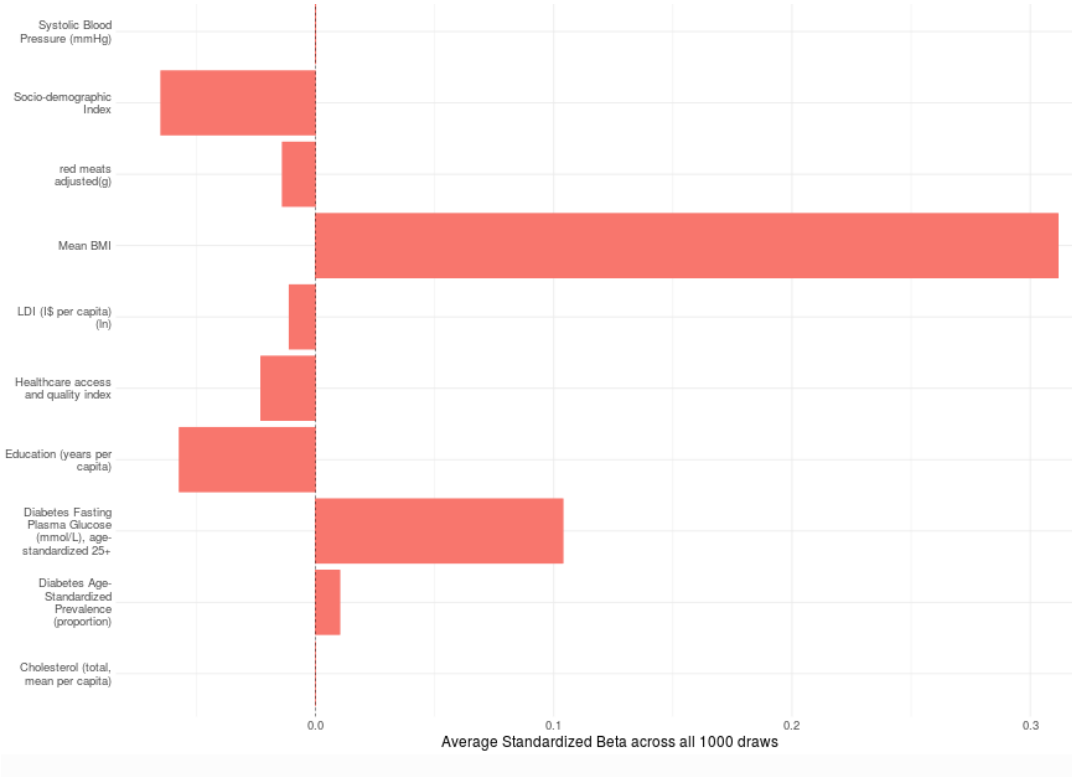

Male, global model

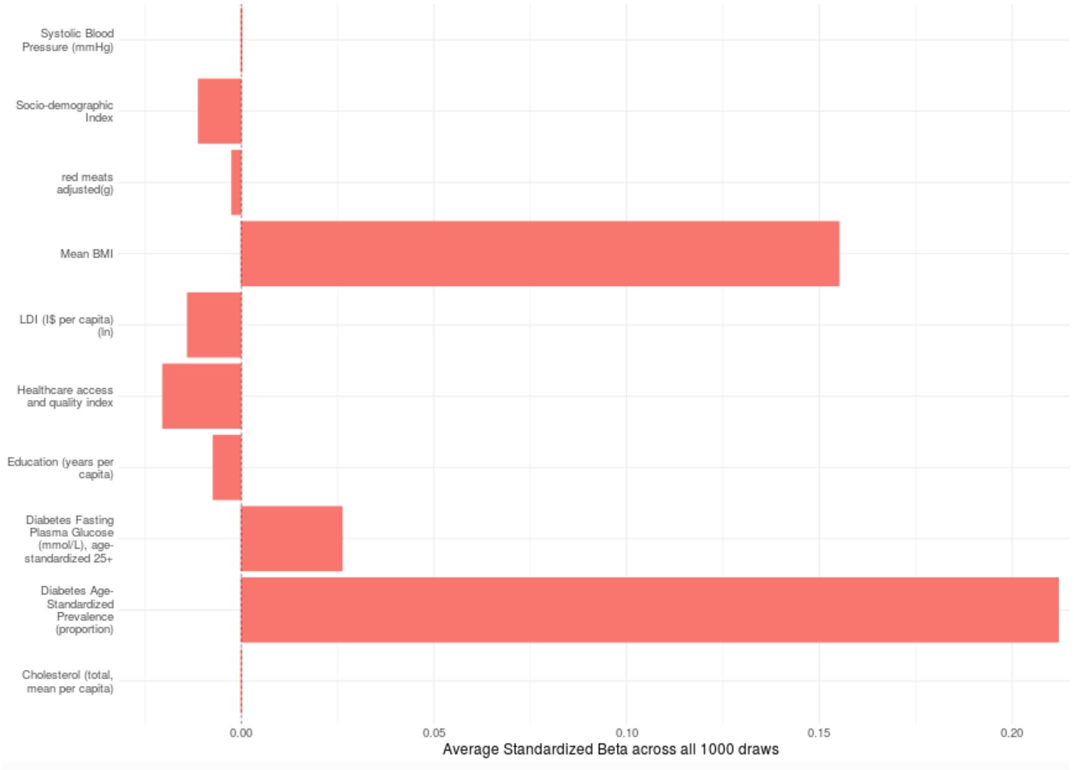

Female, data-rich model

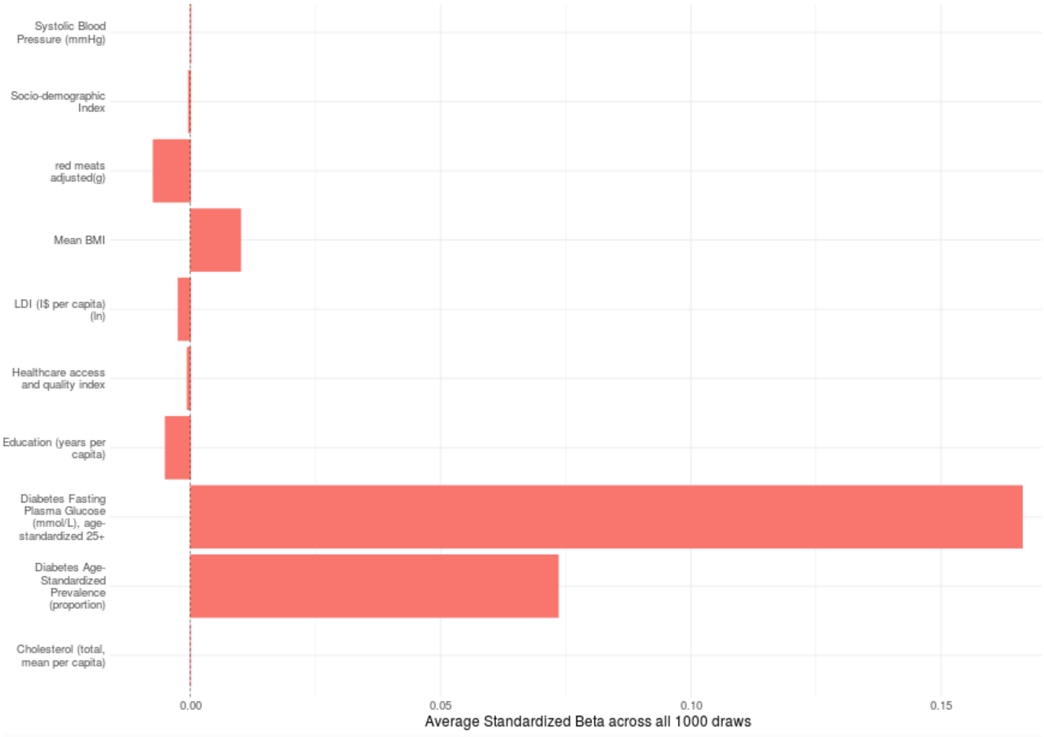

Male, data-rich model

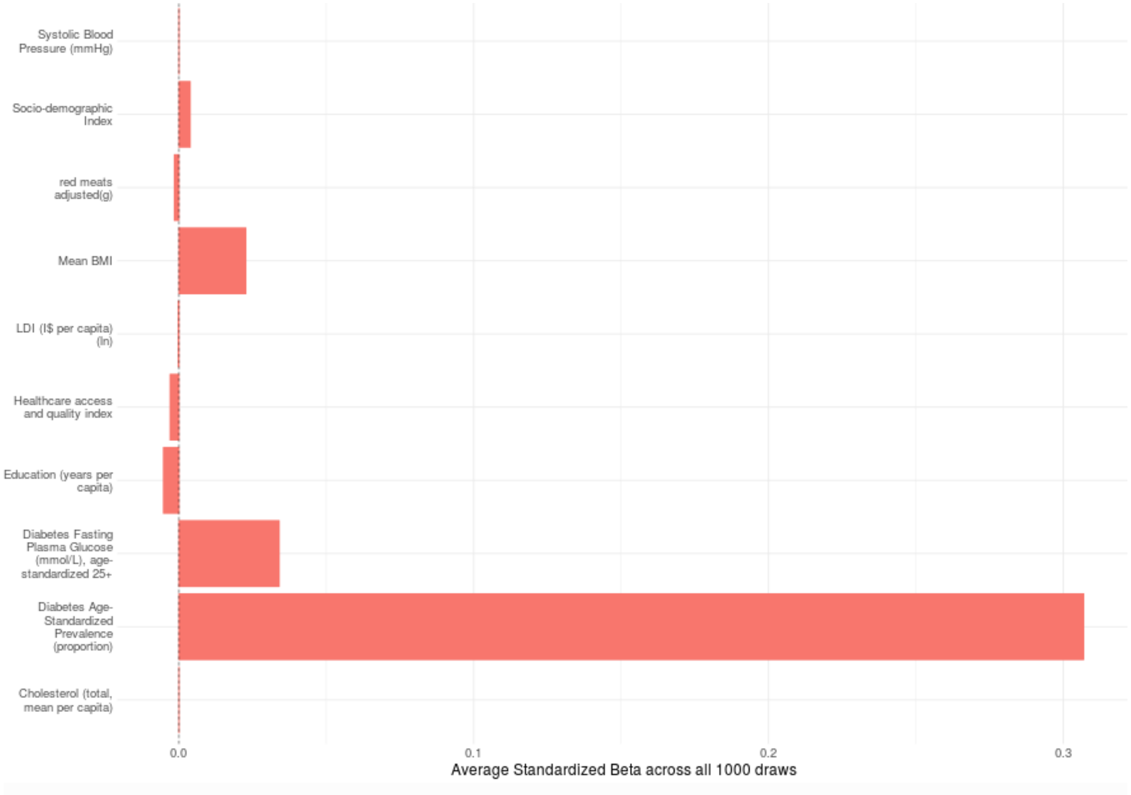

Non-fatal estimation

Chronic Kidney Disease (CKD)

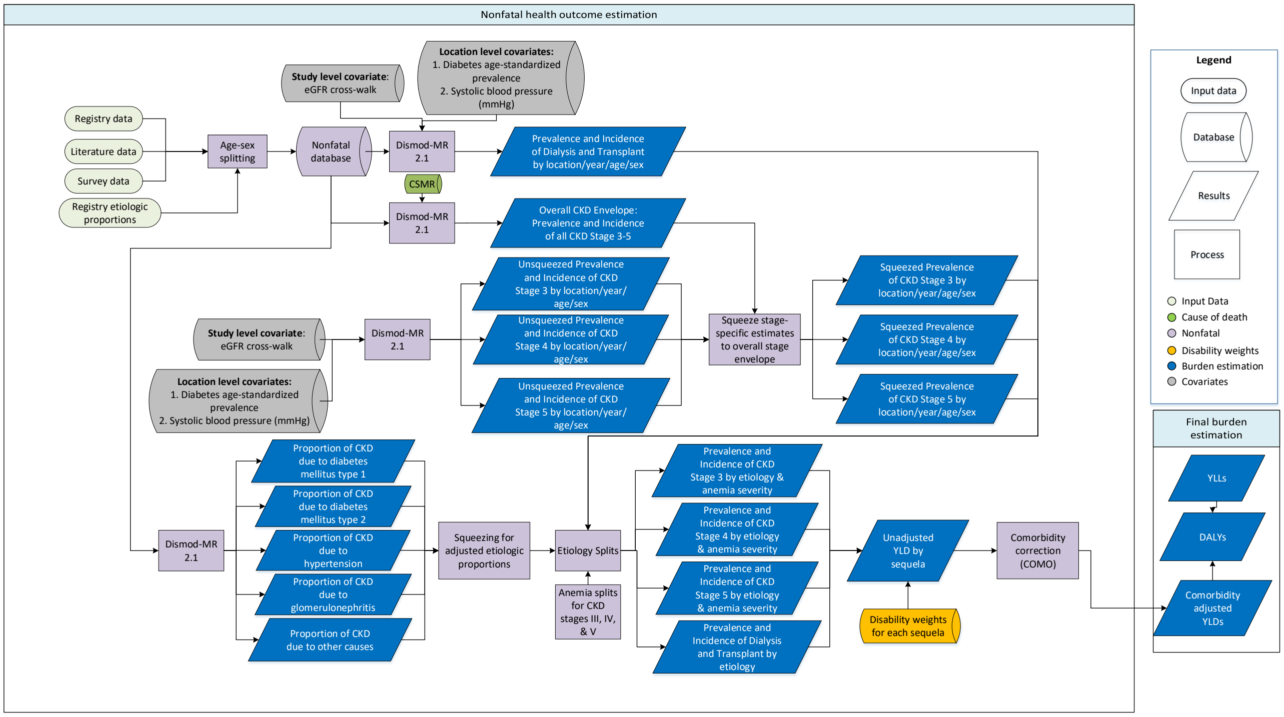

Case definition

Chronic kidney disease (CKD) is defined as elevated urinary albumin to creatinine ratio (ACR), decreased estimated glomerular filtration rate (eGFR), or end-stage kidney disease (ESKD). The GBD study considers six stages of CKD: CKD stages 1-2 (eGFR > 60ml/min/1.73m^2 and ACR > 30 mg/g), CKD stage 3 (eGFR 30-59ml/min/1.73m^2), CKD Stage 4 (eGFR 15-29ml/min/1.73m^2), CKD Stage 5 (eGFR <15ml/min/1.73m^2), ESKD, maintenance dialysis, and renal transplantation.

Input data

Model inputs

For GBD 2010, a systematic review of the prevalence of CKD throughout the world was conducted. This search was updated for GBD 2013, GBD 2015, and GBD 2016. For GBD 2017, this literature search was repeated using PubMed search terms ((((("chronic kidney disease"[Title/Abstract]) AND prevalen*[Title/Abstract]) AND ("1980/1/1"[Date - Publication] : "3000"[Date - Publication])) NOT ((animals[MeSH] NOT humans[MeSH])))).

The exclusion criteria were:

1. Studies clearly not representative of the national population
2. Studies that did not provide primary data on epidemiological parameters, eg, a commentary piece
3. Studies of a specific aetiology of CKD

This literature search was augmented by identification of population-based surveys that measured renal function. For maintenance dialysis and renal transplantation, data were largely obtained from renal registry reports.

Supplemental figure S1 shows the geographical coverage of input data for non-fatal CKD models by CKD stage and estimation year in GBD 2017. There are large disparities in the availability of appropriate, population-representative data across world regions and by stage. Data on RRT (kidney transplantation and dialysis) is much more readily available than data on non-RRT dependent CKD. Sub-Saharan Africa, Latin America and the Caribbean, Eastern Europe, and Central Asia lack epidemiological data on CKD occurrence apart from select RRT registries and RRT facility censuses. Oceania lacks data on the non-fatal burden of CKD altogether. The quality and reliability of CKD burden estimates relies heavily on the availability of epidemiological data. The subsequent supplemental materials detail the GBD approach to leveraging available data to fill in gaps in knowledge using statistical estimation frameworks; however, it should be recognized that in some cases estimates are informed by very few primary data sources.

Supplemental Figure S1 - Non-fatal CKD data availability

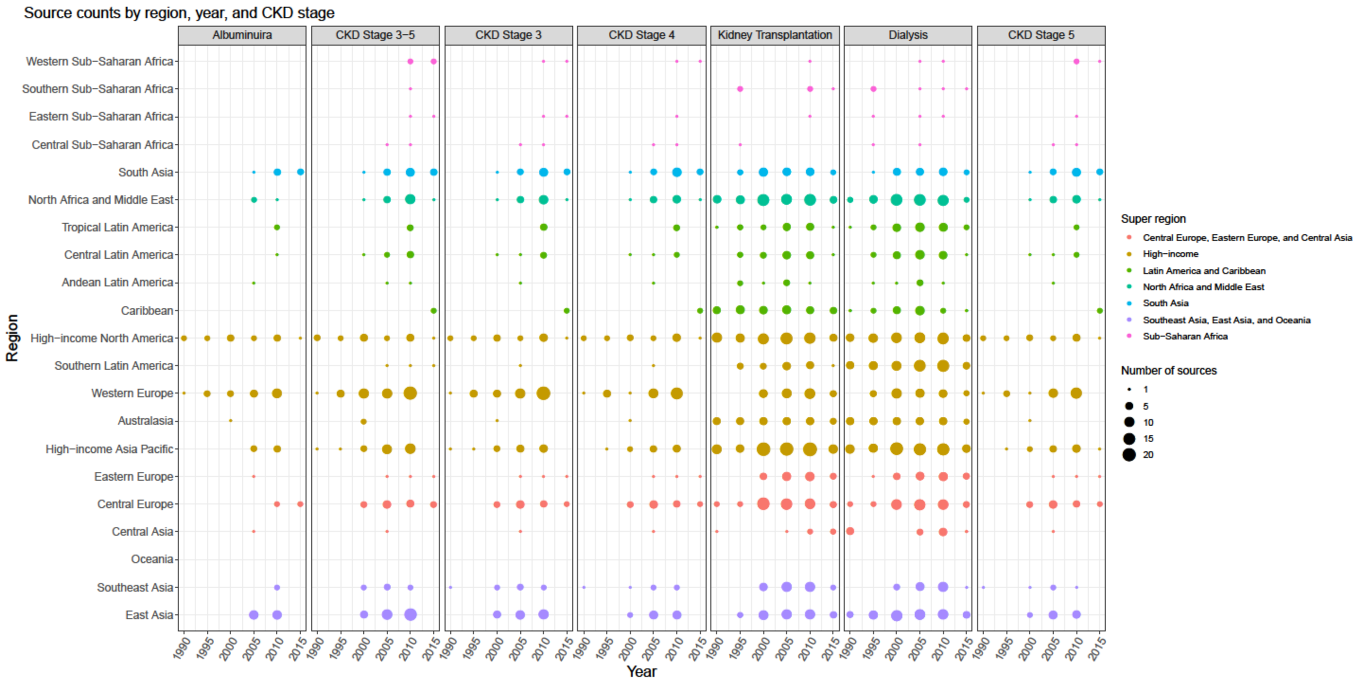

Severity splits & disability weights

Estimates of prevalence and incidence are split using CKD aetiology proportion models, resulting in CKD estimates by stage and aetiology. Then a portion of each aetiology split for CKD stages 3, 4, and 5 is attributed a disability weight associated with mild, moderate, or severe anaemia.[2]

Table S2 - CKD sequelae and disability weights

Sequela	Lay description	Disability weight (95% CI)
CKD stage 1-2	Asymptomatic	--
CKD stage 3 without anaemia	Asymptomatic	--
CKD stage 3 with mild anaemia	Feels slightly tired and weak at times, but this does not interfere with normal daily activities.	0.004 (0.001–0.008)
CKD stage 3 with moderate anaemia	Feels moderate fatigue, weakness, and shortness of breath after exercise, making daily activities more difficult.	0.052 (0.034–0.076)
CKD stage 3 with severe anaemia	Feels very weak, tired, and short of breath, and has problems with activities that require physical effort or deep concentration.	0.149 (0.101–0.21)
CKD stage 4 without anaemia	Tires easily, has nausea, reduced appetite, and difficulty sleeping.	0.104 (0.07–0.147)
CKD stage 4 with mild anaemia		0.108 (0.072–0.151)
CKD stage 4 with moderate anaemia		0.15 (0.103–0.207)
CKD stage 4 with severe anaemia		0.237 (0.165–0.324)
CKD stage 5 without anaemia	Has lost a lot of weight and has constant pain. The person has no appetite, feels nauseated, and needs to spend most of the day in bed.	0.569 (0.389–0.727)
CKD stage 5 with mild anaemia		0.570 (0.391–0.727)
CKD stage 5 with moderate anaemia		0.591 (0.414–0.743)
CKD stage 5 with severe anaemia		0.631 (0.456–0.782)
End-stage renal disease, on dialysis	Is tired and has itching, cramps, headache, joint pains, and shortness of breath. The person needs intensive medical care every other day lasting about half a day.	0.571 (0.397–0.725)
End-stage renal disease, with kidney transplant	Sometimes feels tired and down, and has some difficulty with daily activities.	0.024 (0.014–0.039)

Aetiology proportion models are informed by data from end-stage kidney disease registries and the Geisinger Health System in Pennsylvania provided by the Chronic Kidney Disease Prognosis Consortium (CKDPC) at the Johns Hopkins Bloomberg School of Public Health. Aetiologies included in the GBD study include diabetes mellitus type 1, diabetes mellitus type 2, hypertension, glomerulonephritis, and other and unknown causes.

Modelling strategy

CKD Stage Models

We ran a DisMod-MR 2.1 model to produce estimates by age, sex, year, and country for each stage of CKD. To account for progression of individuals from stage 3 to stage 4, and stage 4 to stage 5, we informed remission for stages 3 and 4. We calculated remission for CKD stage *c*, as the ratio of incidence of *c* to prevalence of stage *c-1* for the same location (l), year (y), age (a), sex (s) group.

$$Remission_{c,l,y,a,s} = \frac{Incidence_{c,l,y,a,s}}{Prevalence_{c-1,l,y,a,s}}$$

We added these remission estimates as data to CKD stage 4 and stage 5 models as well as the renal transplant model (where *c* is renal transplantation and *c-1* is maintenance dialysis). Remission was set to 0 for stage 5 and the excess mortality parameter was used to account for progression to end-stage renal disease and mortality due to CKD stage 5. Bounds on excess mortality were informed using a meta-analysis of survival analyses of individuals with untreated CKD stage 5.

We used data from sources reporting the prevalence of CKD stage 3, 4, and 5 combined (eGFR < 60 ml/min/1.73m^2) to model the prevalence of CKD stage 3-5. We ran a DisMod-MR 2.1 model to produce estimates by age, sex, year, and country for aggregate CKD stage 3-5. We included cause-specific mortality rate (CSMR) estimates from our cause of death modelling in the CKD stage 3-5 model and matched these data with prevalence data points for the same geography-year to estimate expected values of the excess mortality rate (by dividing CSMR by prevalence).

In order to enforce more consistency between stage models, prevalence of CKD stage 3, 4, and 5 were then scaled to sum to the prevalence and incidence of the stage 3-5 CKD model, at the gender-, age-, and country-matched level. CKD stage 3, 4, and 5 models also included predictive covariates for age-standardised prevalence of diabetes and mean systolic blood pressure.

A description of priors and covariates included in each model can be found in the table below:

Table S3 - CKD DisMod model covariate priors

	Priors (min,max)	Study-level covariate	Country-level covariate
CKD stage 3	Remission (0, 0.75)<br>Excess mortality (0, 0.05)	Adjust for estimating equation	Diabetes age-standardised prevalence
			Mean systolic blood pressure
CKD stage 4	Remission (0, 0.75)<br>Excess mortality (0, 0.05)	Adjust for estimating equation	Diabetes age-standardised prevalence
			Mean systolic blood pressure
CKD stage 5	Remission (0, 0)<br>Incidence (0, 0.001), age 0-20<br>Excess mortality (0.29, 0.54)	Adjust for estimating equation	Diabetes age-standardised prevalence
			Mean systolic blood pressure
CKD stage 3-5	Remission (0, 0)<br>Excess mortality (0, 0.54)	Adjust for estimating equation	Diabetes age-standardised prevalence
			Mean systolic blood pressure

eGFR reported for children was estimated using the Schwartz equation as the reference among the paediatric population.[1] We included a fixed effect on data reporting glomerular filtration rate (GFR) estimated with the Modification of Diet in Renal Disease (MDRD) formula to adjust to data reporting prevalence with the CKD-EPI equation. Priors on the MDRD-CKD-EPI fixed effect were informed using a meta-analysis of studies reporting prevalence of CKD by stage using both the MDRD and CKD-EPI equations. Betas and exponentiated values for this crosswalk are shown in the table below:

Table S4 - CKD DisMod study-level covariate betas

	Study covariate	Parameter	beta	Exponentiated beta
Stage 3	eGFR calculated with MDRD equation	Prevalence	0.27 (0.26–0.28)	1.31 (1.30–1.32)
Stage 4	eGFR calculated with MDRD equation	Prevalence	-0.011 (-0.026 to 0.011)	0.99 (0.97–1.01)
Stage 5	eGFR calculated with MDRD equation	Prevalence	-0.091 (-0.14 to -0.034)	0.91 (0.87–0.97)
Stage 3-5	eGFR calculated with MDRD equation	Prevalence	0.24 (0.23–0.25)	1.27 (1.26–1.28)

For the CKD stages 1-2 model, we included a fixed effect on data defining CKD stages 1-2 using ACR thresholds other than 30 mg/g. Priors on the ACR-threshold fixed effect were derived from analysis of the NHANES dataset. For each year of NHANES data, we calculated the prevalence of CKD stages 1-2 using both the reference definition of eGFR > 60ml/min/1.73m^2 with ACR > 30 mg/g and each alternate definition of eGFR > 60ml/min/1.73m^2 with ACR > 17mg/g, 20 mg/g, and 25 mg/g (as these were the threshold values used in extracted studies). For each alternate threshold, we then ran a linear regression where the dependent variable was CKD stages 1-2 prevalence calculated using the reference threshold

and the independent variable was CKD stages 1-2 prevalence calculated using the alternate threshold. Beta values for this crosswalk are shown in the table below.

Table S5 – Crosswalk betas for alternate ACR thresholds

ACR threshold	beta
17 mg/g	2.08 (1.53–2.64)
20 mg/g	1.66 (1.22–2.10)
25 mg/g	1.31 (1.11–1.50)

In order to obtain an appropriate age-pattern with which to age-split dialysis input data, we first ran a DisMod-MR 2.1 model containing only age-specific dialysis data. We then used age-pattern by super-region from this model to age-split dialysis input data, thereby allowing for variation in the age-pattern by location. After age-splitting, we ran a model on all processed data, including age-split data and age-specific data, to obtain final estimates of dialysis incidence and prevalence by location, year, age, and sex. Remission data for dialysis were calculated as the ratio of the incidence of renal transplantation to prevalence of dialysis at the gender-, age-, and country-matched level.

Given the paucity of age-specific data on renal transplantation, we did not age-split input data for this model. Socio-demographic Index was used as a covariate on incidence. Betas and exponentiated values for SDI are as follows:

Table S6 - ESRD transplant country-level covariate betas

	Study covariate	Parameter	beta	Exponentiated beta
ESRD Transplant	Socio-demographic Index	Incidence	1.78 (1.13–2.00)	5.91 (3.08–7.38)

CKD aetiology proportion models

For GBD 2017 we implemented stage-specific aetiology splits to allow for differential aetiological composition of CKD across stages for disease progression. In order to obtain age-sex-stage-specific aetiology proportions, we utilised data from the Geisinger Health System in Pennsylvania to identify patients with CKD. Analysis of this dataset was conducted by the CKDPC. For each individual with CKD, we scanned their history of recorded ICD codes to identify ICD codes for primary renal diseases. We used this information to map individuals to GBD aetiologies by stage of CKD; individuals with CKD but with no history of a primary renal disease ICD code were classified as having CKD of unknown aetiology. We ran a multinomial logistic regression including sex and a non-linear term for age to predict the probability of each aetiology by age and sex for each stage of CKD (1-2, 3, and 4-5 CKD combined). For each stage, aetiology, age, and sex, we converted this probability into the proportion of CKD due to the given aetiology, and applied these proportions to the prevalence of CKD for the same stage, age, and sex category to estimate the prevalence of each stage of CKD by aetiology, age, and sex. The ICD to GBD aetiology map utilised in this analysis is as follows:

Table S7 - GBD CKD aetiology categories and corresponding ICD 9/10 codes

CKD aetiology	ICD 9 Codes	ICD 10 Codes
Type 1 diabetes	250.41, 250.43	E10.2, E10.21, E10.22, E10.29
Type 2 diabetes	250.40, 250.42	E11.2, E11.21, E11.22, E11.29
Glomerulonephritis	581, 581.0, 581.1, 581.2, 581.3, 581.8, 581.81, 581.89, 581.9, 582, 582.0, 582.1, 582.2, 582.4, 582.8, 582.81, 582.89, 582.9, 583, 583.0, 583.1, 583.2, 583.4, 583.6, 583.7, 583.8, 583.81, 583.89, 583.9	N02, N02.0, N02.1, N02.2, N02.3, N02.4, N02.5, N02.6, N02.7, N02.8, N02.9, N03, N03.0, N03.1, N03.2, N03.3, N03.4, N03.5, N03.6, N03.7, N03.8, N03.9, N04, N04.0, N04.1, N04.2, N04.3, N04.4, N04.5, N04.6, N04.7, N04.8, N04.9, N05, N05.0, N05.1, N05.2, N05.3, N05.4, N05.5, N05.6, N05.7, N05.8, N05.9, N06, N06.0, N06.1, N06.2, N06.3, N06.4, N06.5, N06.6, N06.7, N06.8, N06.9
Hypertension	403, 403.0, 403.00, 403.01, 403.1, 403.10, 403.11, 403.6, 403.9, 403.90, 403.91, 404, 404.0, 404.00, 404.01, 404.02, 404.03, 404.1, 404.10, 404.11, 404.12, 404.13, 404.9, 404.90, 404.91, 404.92, 404.93	I12, I12.0, I12.1, I12.2, I12.9, I13, I13.0, I13.1, I13.10, I13.11, I13.2, I13.9
Other	589, 589.0, 589.1, 589.9, 753.0, 753.1, 753.10, 753.11, 753.12, 753.13, 753.14, 753.15, 753.16, 753.17, 753.19, 753.2, 753.20, 753.21, 753.22, 753.23, 753.29, 753.3, 283.11, 710.0, 753.0, 753.21, 753.22, 753.29	N07, N07.0, N07.1, N07.2, N07.3, N07.4, N07.5, N07.6, N07.7, N07.8, N07.9, N08, N08.0, N08.1, N08.2, N08.3, N08.4, N08.5, N08.8, N15.0, Q61, Q61.0, Q61.00, Q61.01, Q61.02, Q61.1, Q61.11, Q61.19, Q61.2, Q61.3, Q61.4, Q61.5, Q61.8, Q61.9, Q62, Q62.0, Q62.1, Q62.10, Q62.11, Q62.12, Q62.2, Q62.3, Q62.31, Q62.32, Q62.39, Q62.4, Q62.5, Q62.6, Q62.60, Q62.61, Q62.62, Q62.63, Q62.69, Q62.7, Q62.8, D59.3, M31.31, M32.14, M32.15, N11.9, N13.70, N13.8, Q60.2, Q63.8, N14.0, N14.1, N14.3, N25.89, N26.9, N28.0

In order to maintain consistency between GBD estimates of type 1 diabetes prevalence estimates and CKD due to type 1 diabetes prevalence estimates and generalise the results of the Geisiger analysis to all locations, we performed a location-specific correction for the proportion of CKD due to type 1 and type 2 diabetes, as type 1 diabetes makes up a much larger proportion of total diabetes in the United States than it does in other locations. For each diabetic subtype (e) for a given location (l), age (a), and sex (g) the ratio of subtype-specific diabetes prevalence to total diabetes prevalence (r) is calculated as:

$$r_{e,l,a,g} = \frac{prevalence_{e,l,a,g}}{prevalence_{dm1,l,a,g} + prevalence_{dm2,l,a,g}}$$

Where e represents either type 1 or type 2 diabetes, dm1 represents type 1 diabetes, and dm2 represents type 2 diabetes.

This ratio is used to adjust the proportion of CKD due to a given diabetic subtype (p) for a given CKD stage (s), l, a, and g by scaling the predicted proportion of CKD due to that subtype (k) by the ratio of total DM due to e in l to the ratio of total DM due to e in the United States (USA).

$$p_{s,e,l,a,g} = k_{s,a,g} \times \frac{r_{e,l,a,s}}{r_{e,USA,a,s}}$$

The stage-specific approach utilised to estimate the prevalence of CKD stages 1-2, 3, 4, and 5 is limited by the use of data from a single geographical region.

For end-stage renal disease on dialysis and end-stage renal disease after transplant, we ran DisMod-MR 2.1 models to obtain estimates of proportions for each subtype by location, year, age, and sex. Data for CKD due to overall DM were more widely available than data by type of DM. Models for the proportion of CKD due to hypertension and diabetes included covariates for mean systolic blood pressure and the age-standardised prevalence of diabetes, respectively. Coefficient values from these models are as follows:

Table S8 - CKD aetiology proportion model country-level covariate betas

Model	Covariate	Value	Exponentiated
CKD proportion due to diabetes mellitus	Diabetes age-standardised prevalence	0.72 (0.66–0.78)	2.05 (1.93–2.18)
CKD proportion due to hypertension	Mean systolic blood pressure	0.034 (0.00076–0.12)	1.03 (1.00–1.13)

In order to make use of the large number of sources reporting the proportion of ESRD attributable to overall diabetes mellitus, we ran a model for the proportion of ESRD due to overall diabetes mellitus, in addition to models for type 1 and type 2. Proportion of CKD due to DM type 1 and DM type 2 estimates were then scaled to sum to the proportion of overall DM at the gender-, age-, and country-matched level. The results from all subtype-specific models were adjusted so that estimates across the subtypes equalled 1 at each of 1,000 draws. These adjusted proportions were applied to the DisMod models for dialysis and transplant to obtain estimates of each of these entities by aetiology.

Major changes to the chronic kidney disease estimation process for GBD 2017 as compared to previous iterations of the GBD study include addition of CKD stages 1-2 as well as updated aetiological attribution of CKD to diabetes mellitus type 1 and diabetes mellitus type 2 instead of the combined category of all diabetes mellitus, and implementation of a stage-specific aetiology estimation method to capture differences in CKD severity by aetiology.

Risk factor estimation

Modelling strategy

Estimates of exposure to CKD stages 1-2, 3, 4, and 5 were obtained from the GBD 2017 non-fatal burden of disease analysis, which includes stage-specific prevalence estimates at the country level across 23 age groups for both genders.

Relative risks were calculated by the Chronic Kidney Disease Prognosis Consortium, a consortium composed of population-level cohorts with prospective data collection from several countries. YLDs and YLLs for cardiovascular diseases and gout were obtained from the GBD 2017 study for the same geographical, time period, and age groups as detailed above.

Theoretical minimum risk exposure level

The theoretical minimum risk is a diagnosis of CKD stages 1-2, 3, 4, or 5. An ACR above 30 mg/g and eGFR below 60ml/min/1.73m^2 have been demonstrated in the literature to be the thresholds at which increased cardiovascular and gout events occur secondary to impaired kidney function.[2–11]

Outcomes

Outcomes of impaired kidney function included peripheral artery disease, ischaemic heart disease, ischaemic stroke, haemorrhagic stroke, and gout. Peripheral artery disease was defined as ICD codes: 440.21 440.23, 443.9, 38.18, 39.25, 39.29, 39.50, 84.1x, 707.1x, 785.4, I73.8, I73.9. Ischaemic heart disease was defined as myocardial infarction, sudden cardiac death, and coronary revascularisation. Stroke categories included ischaemic and haemorrhagic stroke but excluded subarachnoid haemorrhage. In addition to cardiovascular diseases and gout, chronic kidney disease was attributed to exposure to impaired kidney function.

Relative risk

A two-stage pooled meta-analysis was used to calculate relative risks for ischaemic heart disease, stroke, and peripheral vascular disease. The relative risk of these conditions was first determined within each cohort, and then a pooled analysis of cohort-level relative risks was performed using a random effects meta-analysis approach. Uncertainty intervals largely overlapped for the relative risks of fatal and non-fatal cardiovascular events from impaired kidney function exposure. Thus, we decided to use the relative risks from the combined analysis for fatal and non-fatal cardiovascular outcomes. Gout relative risk was determined by meta-analysis of a literature review performed for GBD 2013. Search terms included "gout" and "chronic kidney disease". Exclusion criteria for search results included special populations, reversal of exposure and outcome categories, or unclear exposure category definition. This search resulted in four eligible studies; no new studies indicated an increased risk of gout with CKD stages 1-2.

Population attributable fraction

We calculated the cardiovascular and gout fatal and non-fatal burden attributable to the categorical exposure to impaired kidney function using the following equation:

$$PAF = \frac{\sum_{i=1}^{n} P_i(RR_i - 1)}{\sum_{i=1}^{n} P_i(RR_i - 1) + 1}$$

Equation 1. PAF based on categorical exposure

where RR_i is the relative risk for exposure level *i*, P_i is the proportion of the population in that exposure category, and *n* is the number of exposure categories.(11)

Supplemental results

Burden by region

There was greater than seven-fold variation in the rate of age-standardised CKD DALYs between the 21 world regions in 2017 (Table S16). Age-standardised DALY rates due to CKD were highest in central Latin America and Oceania, exceeding 1000 DALYs per 100 000, while age-standardised DALY rates due to CKD in Australasia, eastern Europe, and western Europe were less than 200 per 100 000. Changes in CKD burden varied markedly across world regions from 1990 to 2017: central Latin America and high-income North America both experienced a greater than 30% increase in the age-standardised DALY rate due to CKD, while central Europe, east Asia, eastern sub-Saharan Africa, and high-income Asia Pacific saw decreases greater than 30% over this period. There was also considerable heterogeneity in age-standardised CKD YLL rates, which differed more than 11-fold in 2017. Oceania and central Latin America, the two regions with the highest age-standardised CKD DALY rates, also had the highest age-standardised CKD mortality rates in 2017, with values of 45.2 per 100 000 (95% uncertainty interval [UI] 40.0–50.0) and 42.1 per 100 000 (40.8–43.3), respectively, while age-standardised CKD mortality rates were less than 30 per 100 000 in all other world regions. Since 1990, the age-standardised CKD mortality rates in central Latin America, central Asia, and high-income North America have increased 60.9% (52.7–66.2), 60.9% (53.3–68.9), and 57.3% (53.4–61.1), respectively. The burden of non-fatal CKD showed considerably less variation than that of fatal CKD in 2017 and varied around two-fold between the regions with the highest and lowest age-standardised rates of YLDs due to CKD. Eastern Europe had the highest age-standardised prevalence of CKD in 2017 at a value of 12.4% (11.5–13.4); another nine of 21 regions had an age-standardised CKD prevalence of 10% or higher (central Asia, central Latin America, central sub-Saharan Africa, eastern Europe, North Africa and the Middle East, Oceania, southeast Asia, southern sub-Saharan Africa, and western sub-Saharan Africa). Despite the high age-standardised prevalence of CKD in eastern Europe, CKD severity was relatively low, as eastern Europe was in the bottom third of countries in terms of the age-standardised rate of YLDs due to CKD in 2017. The age-standardised rate of YLDs due to CKD was highest in central Latin America (145.2 per 100 000 [108.7–181.4]), indicating that non-fatal CKD severity was most advanced in this regions in 2017. Southern Latin America, central Latin America, high-income Asia Pacific, Australasia, Oceania, and high-income North America all experienced increases in the age-standardised rate of YLDs due to CKD, but the high-income North America region was the only region in which this increase was statistically significant (18.7% [11.3–25.4]).

Supplemental tables

Table S16: DALYs for chronic kidney disease in 2017, and percentage change of age-standardised rates by location, 1990–2017

**Location**	**DALYS (95% UI)**		
	**Count, 2017**	**Age-standardised rate per 100 000, 2017**	**Percentage change in age-standardised rates between 1990 and 2017**
**Global**	**35 814 731 (33 728 153 to 37 983 011)**	**451 (425 to 478)**	**-8.6 (-11.8 to -5.4)**
**Low SDI**	**4 932 212 (4 594 659 to 5 428 622)**	**557 (518 to 619)**	**-23.6 (-29.2 to -16.2)**
**Low-middle SDI**	**9 091 502 (8 523 246 to 9 653 897)**	**670 (627 to 711)**	**-11.1 (-16.6 to -5.5)**
**Middle SDI**	**11 958 104 (11 306 831 to 12 609 371)**	**538 (508 to 567)**	**-5.5 (-9.3 to -2.3)**
**High-middle SDI**	**4 941 791 (4 576 445 to 5 361 147)**	**289 (267 to 312)**	**-22.8 (-25.6 to -20.3)**
**High SDI**	**4 778 528 (4 374 026 to 5 190 465)**	**236 (214 to 260)**	**-4.0 (-6.5 to -1.8)**
**East Asia**	**5 253 827 (4 850 296 to 5 642 757)**	**270 (250 to 290)**	**-34.9 (-39.2 to -31.7)**
China	4 886 884 (4 502 524 to 5 251 697)	264 (244 to 283)	-36.1 (-40.6 to -32.9)
North Korea	126 220 (108 527 to 144 714)	414 (356 to 474)	8.3 (-9.8 to 28.9)
Taiwan (Province of China)	156 087 (141 055 to 170 726)	426 (384 to 466)	-14.8 (-20.6 to -9.2)
**Southeast Asia**	**4 140 493 (3 901 181 to 4 388 126)**	**659 (620 to 697)**	**-12.4 (-17.5 to -7.2)**
Cambodia	64 898 (57 008 to 74 014)	498 (441 to 564)	-37.6 (-46.6 to -27.2)
Indonesia	1 276 806 (1 187 547 to 1 371 179)	531 (494 to 572)	-24.2 (-30.1 to -17.9)
Laos	38 770 (32 229 to 44 684)	727 (616 to 832)	-41.5 (-51.8 to -30.0)
Malaysia	131 923 (119 812 to 147 653)	497 (452 to 552)	-9.4 (-17.9 to 3.5)
Maldives	1 864 (1 671 to 2 054)	571 (517 to 625)	-61.2 (-67.6 to -55.3)
Mauritius	27 516 (25 100 to 29 664)	1 657 (1 513 to 1 784)	46.7 (32.7 to 60.4)

Myanmar	387 886<br>(338 349 to 443 877)	793<br>(695 to 899)	-39.1<br>(-50.5 to -26.0)
Philippines	1 075 356<br>(950 705 to 1 215 095)	1 296<br>(1 151 to 1 463)	99.5<br>(75.6 to 127.9)
Sri Lanka	123 232<br>(104 537 to 142 792)	508<br>(434 to 587)	-18.8<br>(-31.1 to -5.9)
Seychelles	1 089<br>(1 009 to 1 173)	998<br>(924 to 1 074)	13.1<br>(-1.2 to 25.0)
Thailand	533 203<br>(477 167 to 589 709)	565<br>(506 to 623)	-20.7<br>(-29.3 to -10.8)
Timor-Leste	4 898<br>(3 724 to 5 716)	533<br>(417 to 622)	-32.4<br>(-47.2 to -17.6)
Vietnam	467 603<br>(413 016 to 529 111)	496<br>(441 to 560)	-27.4<br>(-37.6 to -15.0)
**Oceania**	**114 549<br>(97 864 to 132 919)**	**1 282<br>(1 114 to 1 459)**	**16.6<br>(-1.4 to 33.8)**
American Samoa	704<br>(626 to 772)	1 523<br>(1 356 to 1 665)	54.6<br>(26.9 to 77.4)
Federated States of Micronesia	1 262<br>(961 to 1 525)	1 564<br>(1 232 to 1 864)	30.9<br>(0.7 to 62.2)
Fiji	8 801<br>(7 800 to 9 877)	1 116<br>(989 to 1 248)	25.2<br>(2.4 to 47.8)
Guam	1 688<br>(1 509 to 1 845)	945<br>(843 to 1 031)	82.7<br>(48.5 to 105.5)
Kiribati	1 042<br>(845 to 1 207)	1 210<br>(991 to 1 394)	23.8<br>(-4.2 to 45.9)
Marshall Islands	702<br>(581 to 828)	1 708<br>(1 429 to 1 994)	25.4<br>(4.8 to 45.8)
Northern Mariana Islands	505<br>(448 to 564)	972<br>(872 to 1 073)	19.3<br>(0.3 to 39.8)
Papua New Guinea	82 606<br>(67 721 to 99 120)	1 290<br>(1 071 to 1 526)	11.4<br>(-8.4 to 34.0)
Samoa	1 535<br>(1 298 to 1 744)	1 056<br>(905 to 1 197)	25.6<br>(2.3 to 48.7)
Solomon Islands	6 107<br>(5 184 to 7 140)	1 346<br>(1 163 to 1 544)	0.9<br>(-19.0 to 25.6)
Tonga	1 009<br>(838 to 1 169)	1 198<br>(997 to 1 386)	35.7<br>(12.2 to 59.8)
Vanuatu	2 276<br>(1 757 to 2 914)	1 144<br>(897 to 1 434)	27.8<br>(-1.6 to 66.0)
**Central Asia**	**372 160<br>(346 366 to 399 539)**	**446<br>(415 to 479)**	**25.6<br>(19.7 to 32.2)**

Armenia	11 756<br>(10 703 to 12 876)	301<br>(273 to 329)	13.1<br>(4.7 to 21.5)
Azerbaijan	42 636<br>(38 211 to 46 811)	435<br>(389 to 477)	28.2<br>(12.6 to 42.1)
Georgia	21 414<br>(19 636 to 23 381)	411<br>(378 to 447)	60.6<br>(48.5 to 73.1)
Kazakhstan	54 959<br>(50 347 to 60 098)	312<br>(285 to 340)	0.9<br>(-4.7 to 7.4)
Kyrgyzstan	20 913<br>(19 323 to 22 722)	380<br>(352 to 415)	2.2<br>(-4.9 to 10.5)
Mongolia	13 560<br>(12 058 to 16 303)	486<br>(436 to 570)	-57.2<br>(-62.4 to -44.2)
Tajikistan	30 136<br>(26 936 to 33 255)	415<br>(370 to 459)	2.0<br>(-7.7 to 10.9)
Turkmenistan	25 217<br>(23 307 to 27 305)	556<br>(515 to 602)	45.5<br>(33.5 to 58.7)
Uzbekistan	151 569<br>(135 799 to 168 310)	545<br>(488 to 603)	49.8<br>(35.0 to 66.8)
**Central Europe**	**392 719**<br>**(361 853 to 422 973)**	**205**<br>**(188 to 222)**	**-30.3**<br>**(-32.7 to -27.8)**
Albania	10 042<br>(8 607 to 11 761)	277<br>(238 to 323)	-25.7<br>(-35.3 to -14.7)
Bosnia and Herzegovina	14 937<br>(13 417 to 16 339)	272<br>(245 to 297)	-15.1<br>(-21.8 to -8.1)
Bulgaria	36 759<br>(33 695 to 39 918)	303<br>(279 to 329)	25.5<br>(16.7 to 34.8)
Croatia	15 973<br>(14 698 to 17 377)	194<br>(177 to 212)	1.6<br>(-4.3 to 8.3)
Czech Republic	30 510<br>(27 396 to 33 762)	165<br>(148 to 183)	-35.4<br>(-39.7 to -31.0)
Hungary	33 811<br>(30 649 to 37 324)	196<br>(177 to 217)	-1.1<br>(-6.8 to 4.7)
Macedonia	8 788<br>(7 857 to 9 676)	280<br>(251 to 308)	-13.4<br>(-20.2 to -5.9)
Montenegro	2 866<br>(2 577 to 3 192)	310<br>(280 to 343)	-12.8<br>(-20.7 to -4.0)
Poland	93 014<br>(82 885 to 103 484)	150<br>(133 to 167)	-50.2<br>(-54.0 to -46.0)
Romania	75 873<br>(69 217 to 82 279)	229<br>(209 to 249)	-36.3<br>(-40.1 to -32.6)
Serbia	47 611<br>(41 622 to 51 739)	314<br>(279 to 341)	-7.6<br>(-14.9 to 0.0)

Slovakia	17 878<br>(16 108 to 19 705)	214<br>(193 to 236)	-38.9<br>(-44.1 to -31.3)
Slovenia	4 657<br>(4 082 to 5 269)	119<br>(103 to 135)	-33.9<br>(-38.9 to -28.9)
**Eastern Europe**	**559 471<br>(499 492 to 623 186)**	**186<br>(167 to 207)**	**-21.7<br>(-24.4 to -18.7)**
Belarus	17 704<br>(15 204 to 20 444)	126<br>(108 to 145)	-34.6<br>(-39.8 to -28.7)
Estonia	5 192<br>(4 592 to 5 860)	215<br>(190 to 243)	43.9<br>(27.0 to 62.0)
Latvia	6 716<br>(5 945 to 7 536)	197<br>(175 to 222)	9.5<br>(-0.8 to 19.9)
Lithuania	7 363<br>(6 501 to 8 328)	152<br>(134 to 172)	-17.9<br>(-24.4 to -11.3)
Moldova	8 770<br>(7 776 to 9 939)	173<br>(153 to 195)	-8.8<br>(-14.3 to -2.7)
Russia	408 861<br>(367 023 to 454 955)	198<br>(178 to 220)	-24.8<br>(-27.7 to -21.7)
Ukraine	104 864<br>(92 653 to 118 154)	164<br>(146 to 183)	-13.3<br>(-18.1 to -8.0)
**High-income Asia Pacific**	**857 558<br>(762 004 to 956 594)**	**217<br>(190 to 245)**	**-32.3<br>(-36.4 to -28.7)**
Brunei	2 359<br>(2 097 to 2 591)	660<br>(592 to 721)	-13.8<br>(-24.1 to -3.9)
Japan	672 531<br>(595 739 to 754 198)	220<br>(189 to 249)	-26.5<br>(-31.1 to -22.3)
South Korea	165 394<br>(146 189 to 184 570)	207<br>(183 to 232)	-44.9<br>(-49.4 to -40.6)
Singapore	17 274<br>(15 405 to 19 184)	256<br>(228 to 283)	-35.9<br>(-40.3 to -31.4)
**Australasia**	**88 330<br>(80 058 to 97 445)**	**192<br>(173 to 213)**	**2.0<br>(-4.8 to 9.0)**
Australia	73 605<br>(65 821 to 81 884)	188<br>(168 to 210)	0.2<br>(-7.6 to 8.2)
New Zealand	14 725<br>(13 595 to 16 026)	211<br>(194 to 231)	11.2<br>(4.4 to 18.3)
**Western Europe**	**1 405 328<br>(1 281 751 to 1 538 820)**	**161<br>(145 to 179)**	**-17.1<br>(-19.8 to -14.2)**
Andorra	137<br>(116 to 159)	108<br>(91 to 125)	-15.8<br>(-24.2 to -6.8)
Austria	36 411<br>(33 453 to 39 561)	204<br>(186 to 224)	44.5<br>(34.7 to 54.6)

Belgium	33 786<br>(30 282 to 37 497)	153<br>(135 to 172)	-29.8<br>(-34.7 to -25.0)
Cyprus	4 505<br>(3 552 to 5 053)	243<br>(195 to 273)	-25.1<br>(-33.2 to -17.3)
Denmark	17 766<br>(16 087 to 19 592)	173<br>(155 to 191)	38.9<br>(29.8 to 50.3)
Finland	11 807<br>(10 306 to 13 608)	111<br>(95 to 130)	-2.6<br>(-9.5 to 4.7)
France	157 543<br>(138 630 to 178 038)	122<br>(105 to 140)	-19.6<br>(-25.4 to -13.9)
Germany	378 579<br>(341 846 to 421 474)	203<br>(182 to 227)	-3.0<br>(-10.8 to 5.4)
Greece	55 392<br>(50 806 to 60 320)	239<br>(218 to 263)	-28.3<br>(-33.0 to -23.4)
Iceland	558<br>(484 to 641)	111<br>(95 to 127)	-7.7<br>(-14.3 to -1.1)
Ireland	10 558<br>(9 389 to 11 811)	151<br>(134 to 170)	-27.8<br>(-33.2 to -22.3)
Israel	36 694<br>(33 581 to 39 959)	330<br>(300 to 360)	-16.6<br>(-22.3 to -10.7)
Italy	208 635<br>(187 393 to 231 058)	149<br>(132 to 168)	-29.6<br>(-34.1 to -24.9)
Luxembourg	1 535<br>(1 359 to 1 720)	165<br>(145 to 185)	-11.9<br>(-18.8 to -4.7)
Malta	1 782<br>(1 623 to 1 952)	222<br>(201 to 244)	-17.1<br>(-23.1 to -11.8)
Netherlands	45 720<br>(40 634 to 51 111)	147<br>(128 to 166)	-3.5<br>(-9.7 to 3.2)
Norway	10 865<br>(9 684 to 12 066)	125<br>(110 to 141)	9.6<br>(5.3 to 13.6)
Portugal	51 171<br>(46 203 to 55 857)	226<br>(202 to 249)	-21.7<br>(-27.2 to -16.8)
Spain	150 816<br>(136 087 to 167 931)	156<br>(139 to 176)	-40.4<br>(-44.8 to -36.0)
Sweden	25 347<br>(22 553 to 28 463)	131<br>(115 to 150)	15.6<br>(8.7 to 22.9)
Switzerland	23 380<br>(20 742 to 26 101)	139<br>(121 to 158)	21.3<br>(12.4 to 31.1)
United Kingdom	140 885<br>(123 995 to 159 945)	124<br>(108 to 143)	-18.3<br>(-21.5 to -15.1)
**Southern Latin America**	**341 061<br>(312 546 to 372 174)**	**430<br>(395 to 469)**	**-14.0<br>(-19.9 to -7.6)**

Argentina	235 273<br>(211 519 to 259 979)	456<br>(410 to 503)	-18.5<br>(-25.4 to -10.5)
Chile	90 123<br>(80 885 to 99 623)	399<br>(359 to 442)	4.0<br>(-4.5 to 12.8)
Uruguay	15 651<br>(14 075 to 17 258)	311<br>(279 to 344)	-6.4<br>(-14.5 to 2.1)
**High-income North America**	**2 029 960<br>(1 873 554 to 2 193 754)**	**364<br>(334 to 396)**	**39.3<br>(35.1 to 43.9)**
Canada	120 110<br>(106 696 to 134 100)	196<br>(173 to 221)	4.5<br>(-2.5 to 11.1)
Greenland	135<br>(112 to 152)	213<br>(178 to 242)	-0.2<br>(-16.3 to 9.9)
United States	1 909 680<br>(1 767 517 to 2 061 880)	385<br>(353 to 418)	42.6<br>(38.0 to 47.7)
**Caribbean**	**319 058<br>(296 867 to 343 364)**	**641<br>(597 to 690)**	**10.5<br>(3.8 to 17.7)**
Antigua and Barbuda	719<br>(662 to 779)	717<br>(662 to 777)	1.9<br>(-6.8 to 10.7)
The Bahamas	2 881<br>(2 645 to 3 153)	735<br>(677 to 801)	6.6<br>(-2.7 to 17.0)
Barbados	2 273<br>(2 059 to 2 508)	532<br>(480 to 585)	2.7<br>(-5.5 to 11.6)
Belize	2 821<br>(2 644 to 2 993)	914<br>(856 to 970)	40.0<br>(27.9 to 53.9)
Bermuda	388<br>(352 to 427)	351<br>(318 to 388)	-24.0<br>(-30.5 to -17.4)
Cuba	63 933<br>(57 709 to 71 358)	379<br>(342 to 422)	15.5<br>(5.4 to 26.9)
Dominica	831<br>(768 to 894)	1 005<br>(930 to 1 084)	27.1<br>(17.6 to 38.0)
Dominican Republic	68 922<br>(57 514 to 78 623)	713<br>(594 to 812)	46.6<br>(19.0 to 69.6)
Grenada	1 229<br>(1 143 to 1 322)	904<br>(840 to 971)	-2.3<br>(-9.4 to 5.5)
Guyana	5 943<br>(5 258 to 6 644)	882<br>(782 to 988)	33.9<br>(19.7 to 49.6)
Haiti	75 670<br>(63 551 to 90 105)	893<br>(754 to 1 059)	-16.6<br>(-29.0 to -1.5)
Jamaica	22 485<br>(19 402 to 25 731)	774<br>(668 to 885)	34.7<br>(15.4 to 55.3)
Puerto Rico	36 771<br>(33 817 to 39 609)	602<br>(554 to 648)	-9.6<br>(-16.1 to -2.9)

Saint Lucia	1 526<br>(1 395 to 1 660)	741<br>(679 to 806)	-7.0<br>(-14.8 to 1.2)
Saint Vincent and the Grenadines	1 066<br>(979 to 1 154)	815<br>(751 to 880)	21.1<br>(11.2 to 31.3)
Suriname	5 872<br>(5 307 to 6 458)	999<br>(905 to 1 094)	24.6<br>(11.2 to 38.3)
Trinidad and Tobago	13 076<br>(11 091 to 15 263)	752<br>(641 to 879)	18.0<br>(0.2 to 37.9)
Virgin Islands	1 180<br>(1 021 to 1 320)	704<br>(614 to 789)	21.2<br>(5.0 to 37.9)
**Andean Latin America**	**345 100<br>(318 993 to 370 673)**	**620<br>(574 to 666)**	**-8.1<br>(-15.5 to -0.5)**
Bolivia	80 578<br>(66 781 to 95 046)	881<br>(736 to 1 041)	-15.0<br>(-30.8 to 2.0)
Ecuador	131 347<br>(120 968 to 142 844)	870<br>(801 to 946)	55.5<br>(41.8 to 69.9)
Peru	133 175<br>(116 377 to 150 398)	424<br>(370 to 480)	-32.2<br>(-40.0 to -22.6)
**Central Latin America**	**2 605 380<br>(2 496 659 to 2 719 204)**	**1 083<br>(1 038 to 1 130)**	**53.9<br>(47.1 to 59.4)**
Colombia	224 601<br>(201 199 to 249 321)	422<br>(378 to 468)	-33.4<br>(-38.9 to -27.0)
Costa Rica	30 845<br>(28 451 to 33 866)	623<br>(575 to 683)	33.8<br>(23.5 to 45.3)
El Salvador	104 457<br>(70 961 to 124 682)	1 818<br>(1 230 to 2 162)	155.7<br>(31.0 to 217.4)
Guatemala	146 751<br>(133 619 to 159 828)	1 185<br>(1 081 to 1 292)	26.2<br>(15.2 to 38.7)
Honduras	24 943<br>(21 149 to 29 076)	376<br>(318 to 439)	7.1<br>(-8.9 to 24.6)
Mexico	1 755 136<br>(1 692 006 to 1 823 517)	1 472<br>(1 420 to 1 529)	93.8<br>(86.1 to 100.6)
Nicaragua	68 235<br>(55 881 to 76 949)	1 351<br>(1 120 to 1 516)	18.6<br>(-9.7 to 35.6)
Panama	24 057<br>(22 159 to 26 029)	609<br>(561 to 659)	27.5<br>(18.2 to 37.4)
Venezuela	226 355<br>(200 470 to 255 884)	779<br>(691 to 878)	39.4<br>(23.2 to 58.9)
**Tropical Latin America**	**1 008 827<br>(950 837 to 1 071 464)**	**434<br>(409 to 461)**	**-13.9<br>(-16.7 to -11.3)**
Brazil	966 247<br>(911 469 to 1 027 652)	426<br>(402 to 453)	-15.7<br>(-18.5 to -13.1)

Paraguay	42 580<br>(35 064 to 49 347)	762<br>(624 to 883)	70.4<br>(39.3 to 103.8)
**North Africa and Middle East**	**2 504 747<br>(2 297 458 to 2 713 390)**	**535<br>(493 to 576)**	**-24.4<br>(-28.8 to -19.6)**
Afghanistan	177 892<br>(150 158 to 209 315)	964<br>(830 to 1 117)	-30.1<br>(-43.4 to 14.8)
Algeria	145 047<br>(129 305 to 162 080)	413<br>(370 to 461)	-18.1<br>(-25.5 to -10.3)
Bahrain	5 115<br>(4 428 to 5 850)	505<br>(447 to 564)	-38.8<br>(-45.6 to -32.2)
Egypt	463 360<br>(407 936 to 521 558)	702<br>(614 to 789)	-7.1<br>(-17.5 to 4.4)
Iran	305 536<br>(277 506 to 332 645)	422<br>(386 to 457)	-9.8<br>(-15.3 to -4.6)
Iraq	181 952<br>(164 449 to 200 671)	612<br>(554 to 671)	-56.4<br>(-62.2 to -50.2)
Jordan	41 171<br>(36 576 to 45 873)	623<br>(556 to 697)	-30.7<br>(-39.7 to -20.4)
Kuwait	9 081<br>(7 634 to 10 676)	279<br>(242 to 322)	-53.5<br>(-57.8 to -49.2)
Lebanon	20 446<br>(17 830 to 23 634)	311<br>(273 to 356)	-29.8<br>(-37.3 to -21.0)
Libya	36 550<br>(31 646 to 41 708)	703<br>(614 to 799)	2.1<br>(-13.4 to 20.3)
Morocco	143 349<br>(123 635 to 165 415)	442<br>(380 to 509)	-10.4<br>(-22.5 to 3.5)
Palestine	20 347<br>(18 654 to 22 196)	704<br>(644 to 764)	-28.5<br>(-38.2 to -16.6)
Oman	11 469<br>(9 578 to 13 279)	447<br>(370 to 518)	-11.2<br>(-25.2 to 4.6)
Qatar	5 813<br>(4 834 to 6 924)	505<br>(417 to 582)	-45.4<br>(-54.0 to -35.3)
Saudi Arabia	139 538<br>(118 212 to 160 650)	677<br>(569 to 766)	-11.6<br>(-26.6 to 7.5)
Sudan	136 865<br>(115 935 to 163 713)	514<br>(443 to 599)	-29.0<br>(-40.0 to -15.3)
Syria	74 590<br>(64 641 to 85 957)	527<br>(459 to 615)	-41.0<br>(-49.6 to -30.6)
Tunisia	44 190<br>(37 887 to 51 037)	372<br>(320 to 428)	-19.7<br>(-29.8 to -8.7)
Turkey	410 466<br>(366 272 to 453 077)	478<br>(428 to 528)	-36.6<br>(-43.3 to -29.2)

United Arab Emirates	40 088 (32 330 to 48 536)	723 (600 to 856)	-1.8 (-19.6 to 19.3)
Yemen	89 544 (72 026 to 110 756)	502 (412 to 606)	-26.2 (-41.8 to -0.8)
**South Asia**	**9 459 473 (8 875 159 to 10 048 869)**	**634 (594 to 673)**	**-9.5 (-17.1 to -2.6)**
Bangladesh	584 734 (518 831 to 648 043)	436 (389 to 483)	-35.8 (-45.8 to -25.2)
Bhutan	4 514 (3 626 to 5 292)	623 (511 to 727)	-33.1 (-46.7 to -17.6)
India	7 323 901 (6 838 792 to 7 825 363)	617 (576 to 659)	-10.9 (-18.2 to -4.4)
Nepal	154 230 (127 235 to 181 929)	650 (537 to 762)	-17.0 (-32.9 to 1.1)
Pakistan	1 392 094 (1 157 811 to 1 656 378)	933 (778 to 1 108)	26.3 (3.7 to 51.6)
**Central sub-Saharan Africa**	**488 460 (429 342 to 543 120)**	**707 (628 to 784)**	**-21.3 (-30.1 to -10.1)**
Angola	112 762 (97 159 to 128 389)	740 (642 to 836)	-28.0 (-39.8 to -6.9)
Central African Republic	26 770 (22 155 to 32 236)	909 (758 to 1 070)	-14.1 (-27.7 to 8.6)
Congo (Brazzaville)	23 777 (19 577 to 28 312)	770 (647 to 891)	-29.4 (-41.3 to -16.6)
Democratic Republic of the Congo	311 185 (264 646 to 356 523)	676 (579 to 770)	-18.9 (-31.5 to -4.0)
Equatorial Guinea	4 839 (3 502 to 6 574)	739 (535 to 986)	-42.1 (-57.3 to -21.1)
Gabon	9 127 (7 753 to 10 553)	769 (660 to 882)	-17.2 (-28.8 to -4.5)
**Eastern sub-Saharan Africa**	**1 370 509 (1 266 975 to 1 484 315)**	**631 (584 to 684)**	**-31.8 (-38.8 to -23.0)**
Burundi	37 853 (31 930 to 45 013)	656 (560 to 768)	-39.7 (-49.0 to -28.8)
Comoros	3 210 (2 787 to 3 697)	627 (546 to 719)	-32.5 (-43.0 to -20.0)
Djibouti	4 891 (3 727 to 6 411)	709 (551 to 907)	-7.5 (-28.8 to 22.1)
Eritrea	26 558 (21 515 to 32 004)	803 (664 to 945)	-30.2 (-43.5 to -7.6)
Ethiopia	319 121 (288 800 to 352 499)	582 (526 to 649)	-54.5 (-61.9 to -44.4)

Kenya	162 677<br>(147 176 to 178 487)	599<br>(545 to 661)	-5.3<br>(-11.4 to 1.3)
Madagascar	84 246<br>(71 568 to 98 232)	579<br>(496 to 670)	-24.3<br>(-34.6 to -11.6)
Malawi	65 413<br>(56 846 to 74 551)	658<br>(573 to 745)	-17.0<br>(-33.0 to 26.7)
Mozambique	109 418<br>(93 673 to 130 980)	656<br>(557 to 818)	-14.1<br>(-26.8 to -0.1)
Rwanda	39 979<br>(34 255 to 46 260)	557<br>(477 to 643)	-47.2<br>(-55.1 to -37.8)
Somalia	78 385<br>(60 272 to 102 026)	884<br>(689 to 1 118)	-17.0<br>(-38.9 to 27.6)
South Sudan	48 375<br>(38 445 to 61 058)	871<br>(690 to 1 100)	-11.4<br>(-34.7 to 27.1)
Tanzania	206 964<br>(182 449 to 233 448)	623<br>(553 to 705)	-13.9<br>(-27.5 to 12.0)
Uganda	114 385<br>(98 470 to 130 433)	586<br>(508 to 661)	-17.4<br>(-30.6 to -3.4)
Zambia	68 174<br>(60 102 to 76 806)	742<br>(652 to 830)	-30.1<br>(-40.3 to -13.6)
**Southern sub-Saharan Africa**	**370 703<br>(344 512 to 395 175)**	**604<br>(562 to 643)**	**8.9<br>(0.8 to 15.5)**
Botswana	8 073<br>(7 063 to 9 246)	520<br>(458 to 597)	-6.2<br>(-19.1 to 8.9)
Lesotho	13 666<br>(11 164 to 16 309)	984<br>(811 to 1 165)	38.4<br>(12.5 to 67.7)
Namibia	7 479<br>(6 410 to 8 722)	472<br>(406 to 548)	-27.7<br>(-36.8 to -17.3)
South Africa	271 764<br>(252 143 to 289 359)	571<br>(530 to 609)	3.0<br>(-4.8 to 9.3)
Swaziland (eSwatini)	7 505<br>(5 785 to 9 224)	1 054<br>(822 to 1 283)	20.0<br>(-2.6 to 46.6)
Zimbabwe	62 215<br>(54 214 to 72 269)	731<br>(638 to 842)	47.2<br>(24.9 to 72.8)
**Western sub-Saharan Africa**	**1 787 019<br>(1 597 641 to 2 016 486)**	**616<br>(553 to 699)**	**-26.8<br>(-33.5 to -18.7)**
Benin	53 963<br>(44 623 to 64 374)	743<br>(619 to 880)	-24.6<br>(-36.7 to -11.7)
Burkina Faso	112 250<br>(96 574 to 128 977)	843<br>(739 to 955)	-12.3<br>(-23.7 to 1.9)
Cameroon	132 847<br>(110 253 to 156 104)	770<br>(645 to 907)	-31.1<br>(-42.2 to -19.1)

Cape Verde	2 110<br>(1 888 to 2 359)	430<br>(386 to 481)	2.4<br>(-8.3 to 14.8)
Chad	72 943<br>(63 038 to 85 023)	744<br>(641 to 869)	-17.3<br>(-28.6 to -4.0)
Côte d'Ivoire	124 642<br>(107 601 to 145 841)	773<br>(668 to 910)	-19.9<br>(-32.1 to -6.0)
The Gambia	9 364<br>(7 701 to 11 394)	710<br>(601 to 839)	-21.1<br>(-33.6 to -7.9)
Ghana	153 081<br>(130 817 to 176 421)	698<br>(601 to 793)	6.0<br>(-9.4 to 23.5)
Guinea	62 337<br>(54 731 to 70 742)	800<br>(705 to 913)	-23.5<br>(-33.5 to -11.4)
Guinea-Bissau	11 398<br>(9 499 to 13 698)	998<br>(850 to 1 185)	-34.1<br>(-45.0 to -20.9)
Liberia	21 259<br>(17 746 to 25 522)	715<br>(600 to 839)	-38.1<br>(-47.5 to -27.5)
Mali	106 029<br>(89 495 to 126 272)	705<br>(610 to 819)	-38.2<br>(-46.1 to -28.3)
Mauritania	16 353<br>(13 856 to 19 006)	635<br>(535 to 739)	-41.5<br>(-50.8 to -31.7)
Niger	86 803<br>(70 762 to 105 647)	650<br>(544 to 785)	-41.4<br>(-50.5 to -30.5)
Nigeria	678 032<br>(543 477 to 855 576)	474<br>(375 to 607)	-32.4<br>(-45.0 to -13.7)
Sao Tome and Principe	1 584<br>(1 384 to 1 806)	1 144<br>(996 to 1 303)	-5.8<br>(-18.9 to 10.5)
Senegal	71 046<br>(61 387 to 83 177)	736<br>(641 to 858)	-30.5<br>(-39.7 to -19.8)
Sierra Leone	39 118<br>(33 623 to 45 244)	719<br>(624 to 831)	-30.7<br>(-41.2 to -16.7)
Togo	31 842<br>(26 928 to 37 512)	651<br>(557 to 757)	-26.2<br>(-38.2 to -13.6)

Table S17: Socio-demographic Index groupings by geography, 2017

Geography	SDI Quintile
Global	
Central Europe, Eastern Europe, and Central Asia	
Central Asia	
Armenia	High-middle SDI
Azerbaijan	High-middle SDI
Georgia	High-middle SDI
Kazakhstan	High-middle SDI
Kyrgyzstan	Low-middle SDI

Mongolia	Middle SDI
Tajikistan	Low-middle SDI
Turkmenistan	Middle SDI
Uzbekistan	Middle SDI
Central Europe	
Albania	Middle SDI
Bosnia and Herzegovina	High-middle SDI
Bulgaria	High-middle SDI
Croatia	High SDI
Czech Republic	High SDI
Hungary	High-middle SDI
Macedonia	High-middle SDI
Montenegro	High-middle SDI
Poland	High SDI
Romania	High-middle SDI
Serbia	High-middle SDI
Slovakia	High SDI
Slovenia	High SDI
Eastern Europe	
Belarus	High-middle SDI
Estonia	High SDI
Latvia	High SDI
Lithuania	High SDI
Moldova	Middle SDI
Russia	High-middle SDI
Ukraine	High-middle SDI
High-income	
Australasia	
Australia	High SDI
New Zealand	High SDI
High-income Asia-Pacific	
Brunei	High SDI
Japan	High SDI
South Korea	High SDI
Singapore	High SDI
High-income North America	
Canada	High SDI
Greenland	High-middle SDI
USA	High SDI
Southern Latin America	
Argentina	High-middle SDI
Chile	High-middle SDI
Uruguay	High-middle SDI
Western Europe	

Andorra	High SDI
Austria	High SDI
Belgium	High SDI
Cyprus	High SDI
Denmark	High SDI
Finland	High SDI
France	High SDI
Germany	High SDI
Greece	High SDI
Iceland	High SDI
Ireland	High SDI
Israel	High-middle SDI
Italy	High SDI
Luxembourg	High SDI
Malta	High SDI
Netherlands	High SDI
Norway	High SDI
Portugal	High-middle SDI
Spain	High SDI
Sweden	High SDI
Switzerland	High SDI
United Kingdom	High SDI
England	High SDI
Northern Ireland	High SDI
Scotland	High SDI
Wales	High SDI
Latin America and Caribbean	
Andean Latin America	
Bolivia	Low-middle SDI
Ecuador	Middle SDI
Peru	Middle SDI
Caribbean	
Antigua and Barbuda	High-middle SDI
The Bahamas	High-middle SDI
Barbados	High-middle SDI
Belize	Low-middle SDI
Bermuda	High-middle SDI
Cuba	Middle SDI
Dominica	Middle SDI
Dominican Republic	Low-middle SDI
Grenada	Middle SDI
Guyana	Low-middle SDI
Haiti	Low SDI
Jamaica	Middle SDI

Puerto Rico	High-middle SDI
Saint Lucia	Middle SDI
Saint Vincent and the Grenadines	Middle SDI
Suriname	Middle SDI
Trinidad and Tobago	Middle SDI
Virgin Islands	High-middle SDI
Central Latin America	
Colombia	Middle SDI
Costa Rica	Middle SDI
El Salvador	Low-middle SDI
Guatemala	Low-middle SDI
Honduras	Low-middle SDI
Mexico	Middle SDI
Nicaragua	Low-middle SDI
Panama	Middle SDI
Venezuela	Middle SDI
Tropical Latin America	
Brazil	Middle SDI
Paraguay	Middle SDI
North Africa and Middle East	
North Africa and Middle East	
Afghanistan	Low SDI
Algeria	Middle SDI
Bahrain	High-middle SDI
Egypt	Low-middle SDI
Iran	High-middle SDI
Iraq	Low-middle SDI
Jordan	Middle SDI
Kuwait	High-middle SDI
Lebanon	High-middle SDI
Libya	High-middle SDI
Morocco	Low-middle SDI
Palestine	Low-middle SDI
Oman	High-middle SDI
Qatar	High-middle SDI
Saudi Arabia	High-middle SDI
Sudan	Low-middle SDI
Syria	Middle SDI
Tunisia	Middle SDI
Turkey	High-middle SDI
United Arab Emirates	High-middle SDI
Yemen	Low SDI
South Asia	
South Asia	

Bangladesh	Low SDI
Bhutan	Low-middle SDI
India	Low-middle SDI
Nepal	Low SDI
Pakistan	Low-middle SDI
Southeast Asia, East Asia, and Oceania	
East Asia	
China	High-middle SDI
North Korea	Low-middle SDI
Taiwan (Province of China)	High SDI
Oceania	
American Samoa	High-middle SDI
Federated States of Micronesia	Low-middle SDI
Fiji	Middle SDI
Guam	High-middle SDI
Kiribati	Low SDI
Marshall Islands	Low-middle SDI
Northern Mariana Islands	High-middle SDI
Papua New Guinea	Low SDI
Samoa	Low-middle SDI
Solomon Islands	Low SDI
Tonga	Middle SDI
Vanuatu	Low-middle SDI
Southeast Asia	
Cambodia	Low-middle SDI
Indonesia	Middle SDI
Laos	Low-middle SDI
Malaysia	High-middle SDI
Maldives	Middle SDI
Mauritius	High-middle SDI
Myanmar	Low-middle SDI
Philippines	Middle SDI
Sri Lanka	Middle SDI
Seychelles	Middle SDI
Thailand	Middle SDI
Timor-Leste	Low-middle SDI
Vietnam	Middle SDI
Sub-Saharan Africa	
Central sub-Saharan Africa	
Angola	Low-middle SDI
Central African Republic	Low SDI
Congo (Brazzaville)	Low-middle SDI
DR Congo	Low SDI
Equatorial Guinea	Middle SDI

Gabon	Middle SDI
Eastern sub-Saharan Africa	
Burundi	Low SDI
Comoros	Low SDI
Djibouti	Low-middle SDI
Eritrea	Low SDI
Ethiopia	Low SDI
Kenya	Low-middle SDI
Madagascar	Low SDI
Malawi	Low SDI
Mozambique	Low SDI
Rwanda	Low SDI
Somalia	Low SDI
South Sudan	Low SDI
Tanzania	Low SDI
Uganda	Low SDI
Zambia	Low-middle SDI
Southern sub-Saharan Africa	
Botswana	Middle SDI
Lesotho	Low-middle SDI
Namibia	Middle SDI
South Africa	Middle SDI
Swaziland (eSwatini)	Low-middle SDI
Zimbabwe	Low-middle SDI
Western sub-Saharan Africa	
Benin	Low SDI
Burkina Faso	Low SDI
Cameroon	Low-middle SDI
Cape Verde	Low-middle SDI
Chad	Low SDI
Côte d'Ivoire	Low SDI
The Gambia	Low SDI
Ghana	Low-middle SDI
Guinea	Low SDI
Guinea-Bissau	Low SDI
Liberia	Low SDI
Mali	Low SDI
Mauritania	Low-middle SDI
Niger	Low SDI
Nigeria	Low-middle SDI
Sao Tome and Principe	Low-middle SDI
Senegal	Low SDI
Sierra Leone	Low SDI
Togo	Low SDI

GATHER checklist

#	GATHER checklist item	Description of compliance	Reference
**Objectives and funding**			
1	Define the indicators, populations, and time periods for which estimates were made.	Narrative provided in paper and appendix describing indicators, definitions, and populations	Main text (Methods) and appendix
2	List the funding sources for the work.	Funding sources listed in paper	Abstract (Funding), Acklowlegements
**Data Inputs**			
For all data inputs from multiple sources that are synthesised as part of the study:			
3	Describe how the data were identified and how the data were - accessed.	Narrative description of data seeking methods provided	Main text (Methods) and appendix
4	Specify the inclusion and exclusion criteria. Identify all ad-hoc exclusions.	Narrative about inclusion and exclusion criteria by data type provided	Main text (Methods) and appendix
5	Provide information on all included data sources and their main characteristics. For each data source used, report reference information or contact name/institution, population represented, data collection method, year(s) of data collection, sex and age range, diagnostic criteria or measurement method, and sample size, as relevant.	An interactive, online data source tool that provides metadata for data sources by component, geography, cause, risk, or impairment	Online data citation tools: http://ghdx.healthdata.org/gbd-2017
6	Identify and describe any categories of input data that have potentially important biases (e.g., based on characteristics listed in item 5).	Summary of known biases and impact on estimates is discussed in limitations	Appendix and discussion (limitations)
For data inputs that contribute to the analysis but were not synthesised as part of the study:			
7	Describe and give sources for any other data inputs.	Included in online data source tool	http://ghdx.healthdata.org/gbd-2017
For all data inputs:			
8	Provide all data inputs in a file format from which data can be efficiently extracted (e.g., a spreadsheet as opposed to a PDF), including all relevant meta-data listed in item 5. For any data inputs that cannot be shared due to ethical or legal reasons, such as third-party ownership, provide a contact name or the name of the institution that retains the right to the data.	Downloads of input data available through online tools, including data visualisation tools and data query tools; input data not available in tools will be made available upon request	Online data visualisation tools, data query tools, and the Global Health Data Exchange
**Data analysis**			
9	Provide a conceptual overview of the data analysis method. A diagram may be helpful.	Flow diagrams of the overall methodological processes, as well as cause-specific modelling processes, have been provided	Main text (Methods) and appendix
10	Provide a detailed description of all steps of the analysis, including mathematical formulae. This description should cover, as relevant, data cleaning, data pre-processing, data adjustments and weighting of data sources, and mathematical or statistical model(s).	Flow diagrams and corresponding methodological write-ups	Main text (Methods) and appendix

#	GATHER checklist item	Description of compliance	Reference
11	Describe how candidate models were evaluated and how the final model(s) were selected.	Provided in the methodological write-ups	Appendix
12	Provide the results of an evaluation of model performance, if done, as well as the results of any relevant sensitivity analysis.	Provided in the methodological write-ups	Appendix
13	Describe methods for calculating uncertainty of the estimates. State which sources of uncertainty were, and were not, accounted for in the uncertainty analysis.	Appendix	Appendix
14	State how analytic or statistical source code used to generate estimates can be accessed.	Appendix	http://ghdx.healthdata.org/gbd-2017-code
**Results and Discussion**			
15	Provide published estimates in a file format from which data can be efficiently extracted.	GBD 2017 results are available through online data visualisation tools, the Global Health Data Exchange, and the online data query tool	Main text, and online data tools (data visualisation tools, data query tools, and the Global Health Data Exchange)
16	Report a quantitative measure of the uncertainty of the estimates (e.g. uncertainty intervals).	Uncertainty intervals are provided with all results	Main text, appendix, and online data tools (data visualisation tools, data query tools, and the Global Health Data Exchange)
17	Interpret results in light of existing evidence. If updating a previous set of estimates, describe the reasons for changes in estimates.	Discussion of methodological changes between GBD rounds provided in the narrative of the manuscript and appendix	Main text (Methods and Discussion) and appendix
18	Discuss limitations of the estimates. Include a discussion of any modelling assumptions or data limitations that affect interpretation of the estimates.	Discussion of limitations provided in the narrative of the main paper, as well as in the methodological write-ups in the appendix	Main text (Limitations) and appendix

References:

1. Levey, A. S. *et al.* A new equation to estimate glomerular filtration rate. *Ann. Intern. Med.* **150**, 604–612 (2009).
2. Ninomiya, T. *et al.* Chronic kidney disease and cardiovascular disease in a general Japanese population: the Hisayama Study. *Kidney Int.* **68**, 228–236 (2005).
3. Shara, N. M. *et al.* Estimated GFR and Incident Cardiovascular Disease Events in American Indians: The Strong Heart Study. *Am. J. Kidney Dis. Off. J. Natl. Kidney Found.* **60**, 795–803 (2012).
4. Mann, J. F., Gerstein, H. C., Pogue, J., Bosch, J. & Yusuf, S. Renal insufficiency as a predictor of cardiovascular outcomes and the impact of ramipril: the HOPE randomized trial. *Ann. Intern. Med.* **134**, 629–636 (2001).
5. Go, A. S., Chertow, G. M., Fan, D., McCulloch, C. E. & Hsu, C. Chronic kidney disease and the risks of death, cardiovascular events, and hospitalization. *N. Engl. J. Med.* **351**, 1296–1305 (2004).
6. Chronic Kidney Disease Prognosis Consortium *et al.* Association of estimated glomerular filtration rate and albuminuria with all-cause and cardiovascular mortality in general population cohorts: a collaborative meta-analysis. *Lancet Lond. Engl.* **375**, 2073–2081 (2010).
7. De Graauw, J., Chonchol, M., Poppert, H., Etgen, T. & Sander, D. Relationship between kidney function and risk of asymptomatic peripheral arterial disease in elderly subjects. *Nephrol. Dial. Transplant.* **26**, 927–932 (2011).
8. Wattanakit, K. *et al.* Kidney function and risk of peripheral arterial disease: results from the Atherosclerosis Risk in Communities (ARIC) Study. *J. Am. Soc. Nephrol. JASN* **18**, 629–636 (2007).
9. O'Hare, A. M., Vittinghoff, E., Hsia, J. & Shlipak, M. G. Renal Insufficiency and the Risk of Lower Extremity Peripheral Arterial Disease: Results from the Heart and Estrogen/Progestin Replacement Study (HERS). *J. Am. Soc. Nephrol.* **15**, 1046–1051 (2004).

10. Manjunath, G. *et al.* Level of kidney function as a risk factor for atherosclerotic cardiovascular outcomes in the community. *J. Am. Coll. Cardiol.* **41**, 47–55 (2003).

11. Manjunath, G. *et al.* Level of kidney function as a risk factor for cardiovascular outcomes in the elderly. *Kidney Int.* **63**, 1121–1129 (2003).
